# Dynamics of single enzymes confined inside a nanopore

**DOI:** 10.1039/d5cb00149h

**Published:** 2025-09-18

**Authors:** Nicole Stéphanie Galenkamp, Marco van den Noort, Giovanni Maglia

**Affiliations:** a Chemical Biology, Groningen Biomolecular Sciences & Biotechnology Institute, University of Groningen 9747 AG Groningen The Netherlands Giovanni.maglia@rug.nl; b Division of Physical Chemistry, Department of Chemistry, Lund University P.O. Box 124 22100 Lund Sweden

## Abstract

Enzymes are powerful catalysts that perform chemical reactions with remarkable speed and specificity. Their intrinsic dynamics often play a crucial role in determining their catalytic properties. To achieve a comprehensive understanding of enzymes, a diverse and sophisticated experimental toolbox capable of studying enzyme dynamics at the single-molecule level is necessary. In this review, we discuss nanopore technology as an emerging and powerful platform in single-molecule enzymology. We demonstrate how nanopores can be employed to probe enzyme dynamics in real-time, and we highlight how these studies have contributed to fundamentally and quantitatively elucidating enzymological concepts, such as allostery and hysteresis. Finally, we explore the potentials and limitations of nanopores in advancing single-molecule enzymology. By presenting the unique possibilities offered by nanopores, we aim to inspire the integration of this technology into future enzymology research.

## Abstract 2

This review discusses nanopore technology as an emerging and powerful platform in single-molecule enzymology, highlighting its previous contributions to the field and exploring its future potential.

## Graphical abstract

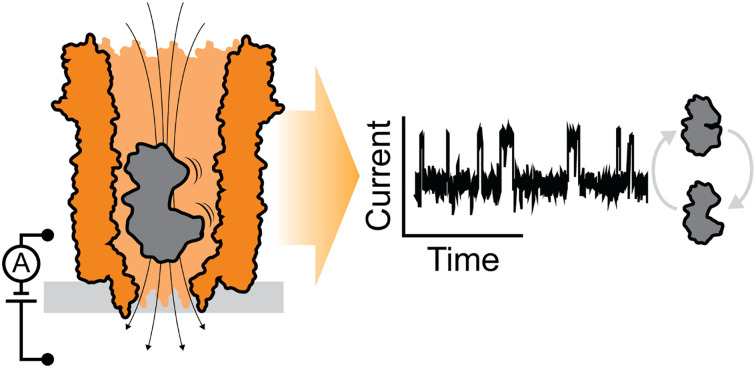

## 1. Introduction

All living organisms use enzymes to catalyze chemical reactions at speeds much greater than would naturally occur without these biological catalysts. The study of enzymes has a longstanding history dating back nearly 150 years. In the nineteenth century, enzymes were first designated by Wilhelm Kühne as non-living substances from biological material capable of performing the chemical activities typically carried out by the organism.^1^ Early breakthroughs introduced foundational concepts such as allostery (1961) and enzyme hysteresis (1970) providing a phenomenological starting point from the enzymology field.^2–6^ Since then, our understanding of enzymes has greatly advanced and the term “enzyme” is now primarily used in reference to biological macromolecules that catalyze chemical reactions.

The availability of structural data, combined with recent advancements in structure prediction software like AlphaFold,^7^ have greatly expanded our understanding of how enzymes' binding pockets facilitate the formation of transition-state geometries and how specific residues within these pockets participate in the chemical reaction.^8–10^ Although the geometry and electrostatic properties of the active site are crucial for its catalytic function, enzyme engineering studies demonstrate that distant mutations significantly influence enzymatic efficiency without altering the overall structure,^11–14^ making a simple sequence–structure–function interpretation of enzymes too simplistic. The structure of enzymes results from the cumulative effect of numerous weak intra- and intermolecular interactions, which can easily break and (re)form, allowing for adopting of different structural states or conformations. Enzymatic properties are often driven by its dynamic behavior.^15^ To connect structure to function, it is necessary to quantify the relative probabilities of the enzyme occupying specific states, as well as the rates at which the enzyme transitions between them. Statistical mechanics provides the mathematical framework for quantifying those probabilities and rates, usually depicted as free-energy landscapes. The timescales of these conformational fluctuations can vary across several orders of magnitude from picosecond-scale rotameric shifts of active site amino acids^16^ to millisecond- or even second-scale global conformational changes. Similarly, catalytic turnover rates span a broad time range. For example, carbonic anhydrase, the fastest known enzyme, interconverts CO_2_ and H_2_O, and H_2_CO_3_ with a turnover number (*k*_cat_) of about 10^6^ s^−1^,^17,18^ while RuBisCO, involved in CO_2_ fixation, catalyzes only a few reactions per second.^19^

**Fig. 1:**
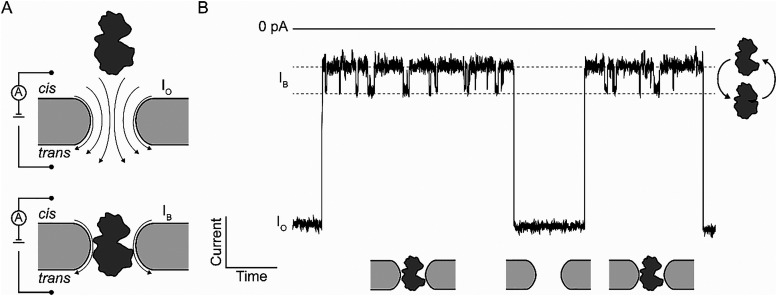
Schematic of recording single-molecule enzyme dynamics within a nanopore. (A) A voltage bias across an insulating membrane creates a current flow through the nanopore (arrows; *I*_O_). An enzyme is trapped within the nanopore, which induces a partial current blockade (*I*_B_). (B) Example of current recordings reporting on the trapping and release or translocation of enzymes. Current fluctuations in the current blockade report on the conformational dynamics of the enzyme, offering valuable insights into the rate and order of enzyme dynamics.

For a comprehensive understanding of enzymatic properties, both conformational information and experimental evidence of the sequence and rates of their interconversion are necessary. To observe structural dynamics ranging from picoseconds to minutes, an extensive experimental toolbox has been developed, with every technique having its own advantages and drawbacks (Table 1). Bulk techniques such as double electron–electron resonance (DEER),^20^ hydrogen–deuterium exchange (HDX-MS)^21^ and NMR spectroscopy^22,23^ provide valuable insights but do average out asynchronous dynamics. In contrast, single-molecule techniques like single-molecule Förster resonance energy transfer (FRET),^23,24^ atomic force microscopy,^25^ and optical tweezers^26^ enable real-time monitoring of the dynamics of individual molecules. In the past decade, a new methodology entered the fray: nanopores were utilized to effectively study substrate-binding kinetics of individual enzymes trapped inside the lumen of a nanopore for seconds to minutes with microsecond time resolution.^27^ At its most basic, a nanopore is a nanometer-scale aperture embedded within an insulated membrane that separates two electrolyte-filled compartments (Fig. 1). Applying a voltage bias across the membrane induces an ionic current flow through the pore. Trapping of an enzyme inside the pore causes a partial current blockage, with the magnitude of current blockage reflecting its conformational state. This allows for continuous recording of global dynamics of single enzymes over extended periods of time.

**Table 1:** Comparison of experimental techniques that report on enzyme dynamics

Technique	Temporal resolution/bandwidth	Advantages	Drawbacks
NMR	ms–h	– Atomic-level resolution	– Bulk measurements
		– Native-like solution conditions	– Requires high sample concentration
			– Expensive and complex instrumentation
DEER (EPR)	μs–ms	– Monitors distance changes	– Bulk measurements
			– Requires protein labeling
			– Requires low temperature
HDX-MS	s–min	– Label-free	– Bulk measurements
		– Near-native solution conditions	– Low spatial resolution
			– Indirect structural read out
smFRET	ns–min	– Monitors distance changes	– Requires protein labeling
		– Single-molecule sensitivity	– Photobleaching
(Optical) tweezers	μs–h	– Real-time monitoring of folding/unfolding	– Requires protein labeling
		– Single-molecule sensitivity	– Force application may affect native enzyme behavior
Atomic force spectroscopy	ms–h	– High spatial resolution	– Requires surface attachment
		– Real-time monitoring of unfolding	
		– Single-molecule sensitivity	
Nanopores	μs–h	– Long recording times	– Indirect signal (current-based)
		– Label-free and real-time	– Signal interpretation can be complex
		– Single-molecule sensitivity	
		– Records global enzyme dynamics	

Nanopores have long been used in nucleic acid sequencing,^28–33^ protein sensing and metabolite analysis,^34–40^ with ongoing effort to adapt them for protein sequencing.^41–45^ Today, even small handheld nanopore devices are commercially available on the market. In enzymology, nanopores have been utilized in three primary approaches. First, (bulk) reaction rates are tracked by nanopores through specific detection of the product or substrate.^46–53^ Second, polymer translocation through nanopores or a nanopore-attached enzyme-binding site allows for measuring reaction speed or binding kinetics of single enzymes which perform their reaction outside of the nanopore.^54–61^ However, this offers limited insights into the enzyme's dynamic behavior. Third, enzymes can be confined within the nanopore lumen, where their dynamic behavior is directly linked to ionic current fluctuations.^27,62–68^ This enables direct monitoring of global enzyme dynamics with high sensitivity.

An overview of these approaches has been reviewed before by us and others.^69,70^ Additionally, other reviews have described how nanopores can serve as tools for investigating protein dynamics.^35,71^ In this review, we specifically focus on single-molecule enzymology inside the nanopore. First, we outline how nanopores can be employed to probe enzyme dynamics, highlighting their capabilities, advantages, and inherent limitations. Second, we discuss longstanding concepts in enzymology and discuss how nanopore technology has facilitated a more detailed and quantitative understanding of these principles. Third, we look to the future, exploring the potential applications of nanopores in advancing single-molecule enzymology. With this review, we aim to inspire enzymologists to embrace the unique possibilities offered by nanopores and encourage them to incorporate this technology into their future research endeavors.

## 2. Nanopores

In a typical nanopore experiment, the two electrolyte-filled reservoirs are known as the *cis* and *trans* compartments (Fig. 1A). Electrodes placed in each compartment apply a voltage across the membrane, creating an ionic current that flows through the nanopore.^72^ The current generated by the nanopore (open pore current, *I*_O_) is indicative of its size and shape, and when an analyte – such as nucleic acids, proteins, or other molecules – is lodged within the nanopore, it causes a blockade in the current (Fig. 1A). This blockade (blocked pore current, *I*_B_) is characterized by the residual current (*I*_res%_ = *I*_B_/*I*_O_ × 100%), which depends on the pore's and analyte's charge, size, and geometry.^73^ The blockade frequency of occurrence corresponds to the analyte's concentration.^28^ Conformational dynamics of the analyte can be inferred from current fluctuations (Fig. 1B). These fluctuations may arise from structural changes in enzymes or ligands, or from binding events, which change the blockade characteristics through altered pore-analyte interactions or different space occupancy inside the pore. Variations in residual current, dwell time, and noise provide valuable insights into conformational transitions and binding events, enabling a deeper understanding of the analyte's behavior under varying environmental conditions.

### 2.1 Equipment

To capture these small current variations with high sensitivity, experiments are conducted using high-resolution equipment, such as patch-clamp amplifiers with a sampling rate of up to 250 kHz. While data acquisition is rapid, the effective temporal resolution is limited by noise and low-pass filtering, typically around 100 μs. Managing this noise is a key challenge, as it originates from various sources, including data acquisition electronics, the access resistance of the system, and the intrinsic noise of the nanopore itself. To reduce external noise, the experiment is conducted inside a Faraday cage, shielding the setup from electromagnetic interference, and environmental vibrations are also usually dumped. Internal sources of noise, such as equipment within the shielded environment, are also carefully controlled. Electrodes made of Ag/AgCl are commonly used due to their chemical stability, low noise, and reliable electrochemical properties, ensuring precise measurements.

The advantage of nanopore recordings is the ability to sense single molecules in real-time without the need for labelling, immobilization or chemical modifications.^74^ Additionally, nanopore measurements are inexpensive, require only a simple set-up and can be used with low reagent volumes in physiological conditions.^28^ Furthermore, nanopores are able to observe native single molecules with no intrinsic limitation of molecular size or observation time. This allows for continuous monitoring of a broad range of dynamic processes, such as conformational dynamics, oligomerization, and unfolding/refolding. Finally, due to their electrical output nanopores can be integrated within electronic devices and are suitable for miniaturization and parallelization technology.^74,75^

### 2.2 Nanopore types

**Fig. 2:**
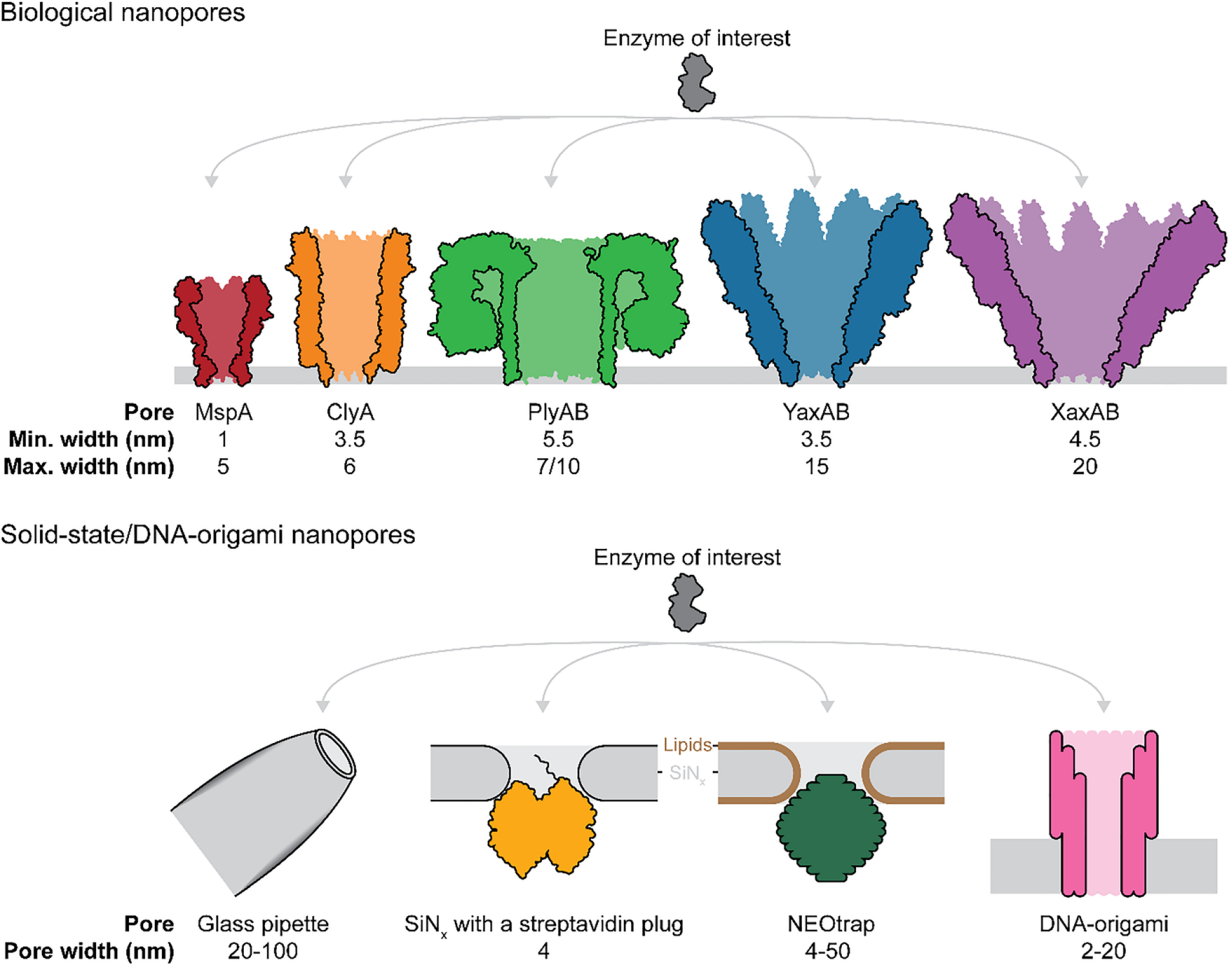
Nanopore types which are suitable for studying single-molecule enzyme dynamics. Top: Cartoon representations biological nanopores MspA (PDB: 1UUN), ClyA (PDB: 6MRT), PlyAB (PDB: 4V2T), YaxAB (PDB: 6EL1), and XaxAB (PDB:6GY6). Bottom: Schematic of measuring proteins with glass, solid-state or DNA origami nanopores. The protein sketches were prepared using ChimeraX (v1.9).

Nanopores can be classified into three main classes: biological nanopores embedded in a lipid bilayer, synthetic nanopores fabricated in solid substrates (*e.g.*, silicon, graphene or glass pipettes),^76–79^ and DNA-origami nanopores^80,81^ (Fig. 2). Biological nanopores, often derived from pore-forming toxins or outer membrane proteins, self-assemble into transmembrane pores upon interaction with membranes or detergents. Examples include pore-forming toxins such as α-hemolysin (αHL),^30,82–84^ fragaceatoxin C (FraC),^85,86^ cytolysin A (ClyA),^66,87^*Yersinia enterocolitica* α-xenorhabdolysin (YaxAB),^37,88^ aerolysin,^89,90^ or outer membrane proteins like *Mycobacterium smegmatis* porin A (MspA),^29,91^*Escherichia coli* outer MPs (OmpG, OmpA, and OmpF),^92,93^ and truncated ferric hydroxamate uptake component A (tFhuA).^40,94^ Some of these nanopores, such as ClyA, FraC and YaxAB, exhibit diverse oligomeric states, producing pores of varying sizes that depend on the number of monomers (Fig. 2). This versatility, combined with their low cost, atomic precision, reproducibility, and the possibility of tuning the pore interior through mutagenesis,^95^ makes them valuable for molecular sensing. However, limitations remain in predicting protein folding and the availability of crystal structures.^96^ Another limitation lies in the necessity of using an inherently unstable lipid membrane, which complicates the fabrication of pore arrays, thus limiting the increase in data-output.

Synthetic or solid-state nanopores are a versatile alternative to biological nanopores due to their customizable size and mechanical, thermal, and chemical stability of the insulating membrane.^77^ This creates potential for device integration, and the ability to form arrays.^97,98^ Coatings are often employed to minimize nonspecific interactions of proteins with the walls of the nanopore.^99–104^ Without such coatings, proteins may bind unpredictably to the nanopore surface, potentially leading to distorted results. While coatings can mitigate these issues, they may also introduce challenges, such as affecting the protein's natural behavior or interfering with the nanopore's sensitivity to subtle conformational changes. Typically, solid-state nanopores are created in freestanding dielectric membranes made from materials like organic polymers, graphene, and silicon using techniques such as focused ion-beam drilling,^76^ electron-beam drilling,^105^ or chemical track-etching.^106^ More recently, nanopores are also formed by controlled dielectric breakdown.^107–109^

Glass nanopores are typically fabricated from quartz or borosilicate glass capillaries with a narrow conical tip, reaching tip diameters of ≤100 nm and are often modified chemically or physically to improve sensitivity and selectivity.^79,110–113^ These nanopores are simple and cost-effective to produce compared to other solid-state nanopores, which often require cleanroom lithography facilities. Common fabrication methods include chemical etching and mechanical pulling of glass capillaries, followed by electrochemical or thermal treatments to control the geometry and size of the tip.^114^ Their strength, optics, and tunable surface make them ideal for precise, noninvasive single-molecule and cell detection.^112,115^ Despite their advantages, they face limitations such as clogging, limited reproducibility, and unstable signals in biological environments.^115–117^

Next to challenges in fabrication and reproducibility, solid-state nanopore analysis is hampered by a lower signal-to-noise ratio, compared to biological nanopores, and fast translocation of folded proteins.^78,118,119^ The latter has been overcome by the nanopore electro-osmotic trap (NEOtrap) (Fig. 2).^120^ The NEOtrap uses a charged object, like a DNA-origami cork, to form a nanocavity in a solid-state nanopore, trapping proteins by inducing a strong electro-osmotic flow. This method extends the observation time of proteins by a factor of one million to one billion compared to free translocation and allowed the monitoring of large conformational changes in Hsp90, including the stabilization of a compact state by ATP or AMP-PNP, and increased structural heterogeneity in the presence of ADP or in the apo form.

DNA origami nanopores represent a third class of nanopores, combining the precision of biological systems with the versatility of synthetic materials.^121,122^ These nanopores are constructed by folding a long single-stranded DNA scaffold into a desired shape using numerous complementary shorter staple strands. This approach allows for precise customization of pore size, shape, and functionality, enabling the creation of nanopores tailored to specific analytes. One of the intrinsic challenges of inserting a DNA nanopore into a lipid bilayer is its negatively charged phosphate backbone.^123^ To overcome this barrier and enhance membrane association, DNA structures are typically decorated with hydrophobic moieties.^124^ The geometry and functionality of artificial DNA nanopores can be designed using molecular engineering tools.^125^ Unlike solid-state nanopores, DNA-based pores offer molecular precision and are easier to chemically modify. Early versions often faced issues with membrane integration, stability, and consistent conductance, requiring further refinement.^126^ Thanks to ongoing improvements in design, direct sensing of IgG antibodies,^127^ 40-kDa dextran–tetramethylrhodamine,^128^ and folded proteins^129^ has been demonstrated, showcasing the potential of DNA nanopores in advanced molecular sensing applications. However, proteins’ conformational changes have yet to be measured.

### 2.3 Enzyme trapping

Once molecules enter a nanopore, they are subjected to several forces. They may interact with the nanopore walls, which either prolongs or shortens their residence time depending on the interaction. These electrostatic and hydrophobic interactions can be modulated by altering the ionic strength or pH of the electrolyte solution,^130^ or by introducing mutations to the nanopore inner surface.^52,66^ In addition, molecules experience an electrophoretic force from the externally applied electric field, and an electro-osmotic flow. The magnitude of the electrophoretic force depends on the charge of the molecule and the voltage bias applied across the nanopore.^131,132^ The electro-osmotic flow arises from the directional movement of hydrated ions along the nanopore walls under an applied potential.^68,133–137^ Its magnitude and direction are affected by factors such as the nanopore's geometry, surface charge, and the electrolyte composition and its concentration in the two chambers.^133,134,137–140^ Furthermore, temperature can influence both electro-osmotic flow and electrophoretic mobility by altering viscosity and ion mobility within the solution.^141,142^ Depending on the surface charge of the nanopore and especially its constriction, the electro-osmotic flow can either augment or counteract the electrophoretic force experienced by molecules, thereby modulating their capture and translocation behaviour.^86^ As a result, predicting the movement and diffusion of molecules through nanopores is challenging due to the interplay of these forces. For small, charged molecules or uniformly charged polymers, electrophoresis tends to be the dominant force influencing their transport.^143^ For proteins and other macromolecules of tens of kilodaltons, electro-osmotic forces become increasingly dominant.^136,144^ In some cases, steric hindrance and entropic effects also significantly contribute, especially for flexible or partially unfolded biomolecules.^142,145^ Molecular dynamics simulations and physical models provide excellent predictions of the analyte's behavior inside the pore, and the direction and magnitude of the forces acting upon it.^37,66,88,146,147^

While it's important to consider that confinement and applied forces in nanopore experiments could influence protein activity, such effects can be evaluated by measuring across different voltages and extrapolating to zero voltage. Supporting studies show that the binding affinity of proteins remains consistent with bulk values suggesting minimal impact within the nanopore environment.^39,63^ Typically, force effects are negligible. Moreover, confinement within the nanopore can be advantageous, as it mimics the crowded intracellular environment where biochemical reactions naturally occur. Thus, studying enzymes in nanopores may better approach physiological conditions compared to conventional single-molecule techniques performed in dilute environments.

Although it is possible to trap a wide range of different proteins in a nanopore, it remains a challenge to optimize signal detection and resolution. Signal quality may be improved by fine-tuning experimental parameters such as bias voltage, ionic strength, salt composition, or pH to enhance ionic current changes. Nanopore engineering further enables improved signal detection and stability. For example, mutating residue E57 in ClyA was shown to reduce electrostatic repulsion to stabilize the trapping of a protease and enhance signal resolution,^66^ while introducing a tryptophan residue in the ClyA variant ClyA-AS prolongs the lifetime of short-lived blockades, increasing the signal output per single-molecule.^52^ Likewise, the DNA-origami structure of NEOtrap has been functionalized with cholesterol moieties to reduce noise and enable long recordings of smaller enzymes.^148^

The enzyme of interest could also be engineered to improve capture and the detection of conformational changes. Adding positively charged tags to negatively charged proteins enhances the dwell time within the nanopore by strengthening electrostatic interactions at the pore constriction.^144^ Additionally, introducing charge dipoles through genetic modification aligns proteins with the nanopore's electric field, minimizing noise and preventing rotational tumbling during enzyme trapping.^149^ However, engineering the enzyme of interest dims the advantage of nanopores as a label-free technique to study natural enzymes.

Potential bottlenecks must be carefully considered during the experimental design phase of a nanopore enzymology experiment, because they may influence the choice of the nanopore system and impact data quality (see Section 4). Firstly, enzyme size, structure, and physicochemical properties greatly affect the experimental outcome. Small enzymes may escape trapping in nanopores with diameters that are too wide, while larger or multimeric ones may not be properly accommodated in pores that are too narrow, preventing enzyme trapping or leading to excessive current fluctuations that complicate the correlation between signal and enzymatic activity. Also, strong interactions between the enzyme and the nanopore or membrane can further hinder reliable measurements. Secondly, signal resolution becomes problematic when ligands bind to the same site or a single ligand binds multiple sites with similar kinetics, making it difficult to distinguish individual events. Thirdly, even when an enzyme can be effectively trapped and signal quality is high, challenges remain in terms of temporal resolution. Enzymatic reactions may occur on timescales of milliseconds or microseconds,^150^ involving rapid conformational changes that may not be accurately captured by nanopore systems, limiting the ability to resolve complete kinetic sequences in real-time. Finally, unlike techniques that directly observe structural changes,^151–154^ nanopore-based methods detect current shifts, which indirectly reflect the enzyme's behavior. As such, interpreting these signals requires careful consideration of the enzyme's conformational dynamics, as current changes may not always correlate with specific conformational dynamics, but also encompasses enzyme movement within the nanopore or changes in physicochemical properties of the enzyme upon substrate binding, complicating the analysis of real-time molecular events.

## 3. Nanopore-based single-molecule enzymology

### 3.1 Non-enzymatic conformational changes

Early efforts to track protein conformational changes using nanopore technology initially concentrated on non-enzymatic processes. Since many enzymes undergo structural changes before or after substrate binding, it became clear that nanopores could also be applied to study enzymatic activities. One of the earliest studies involved investigating the conformational dynamics of alkylation B (AlkB) demethylase and dihydrofolate reductase (DHFR) within the lumen of a ClyA nanopore.^27^ The research demonstrated that even without enzymatic turnover, the presence of ligands could induce stepwise current signals, with the frequency of these signals increasing as ligand concentration rose. Notably, protein variants with reduced affinity for ligands failed to produce these stepwise current blockades, confirming that the signals were specific to ligand binding. Although it remained unclear whether the observed signals were due to different protein conformations or variations in the protein's position, orientation, or interactions within the nanopore, these findings suggested that enzymatic processes could be monitored using nanopore technology. Notably, the binding of NADPH and NADP^+^, which differ by just one hydride ion, produces distinct current block signals when interacting with a DHFR:methotrexate complex inside the nanopore. This indicates that even small differences in protein–ligand complexes can be detected, underscoring the potential of nanopores to monitor intricate enzymatic reactions with high sensitivity.^27^

Another important class of proteins studied in this context includes the periplasmic substrate-binding proteins (SBPs) of ATP-binding cassette (ABC) importer proteins. These SBPs, although not catalytically active, exhibit well-defined conformational changes upon ligand binding, which can be resolved in real time when the protein is trapped inside the nanopore.^39,155^ For instance, SBD1 and SBD2 of the GlnPQ ABC importer from *Lactococcus lactis* in a ClyA nanopore open two-fold slower and closes 100 to 1000 times faster in the presence of the ligand. This suggests that ligand binding triggers a conformational change that results in a faster closing of the protein around the ligand. This ligand-induced acceleration of closing likely reflects a conformational change that enables tight ligand capture and efficient transfer to the transporter, thereby facilitating substrate uptake.^149^ Similar to DHFR, the resolution of nanopore analysis was showcased using the promiscuous maltose-binding protein (MBP) from *E. coli*. MBP binds several oligosaccharides in a variety of conformations with different degrees of closure.^156,157^ It also binds two maltose isomers with different affinity and in a slightly different conformation, which could be resolved in a ClyA nanopore.^158^ These examples collectively highlight the power of nanopore-based sensing for probing the structural dynamics of folded proteins under near-native conditions, setting the stage for more complex enzymology studies.

### 3.2 Rate of conformational dynamics determine/correlate with protein activity

**Fig. 3:**
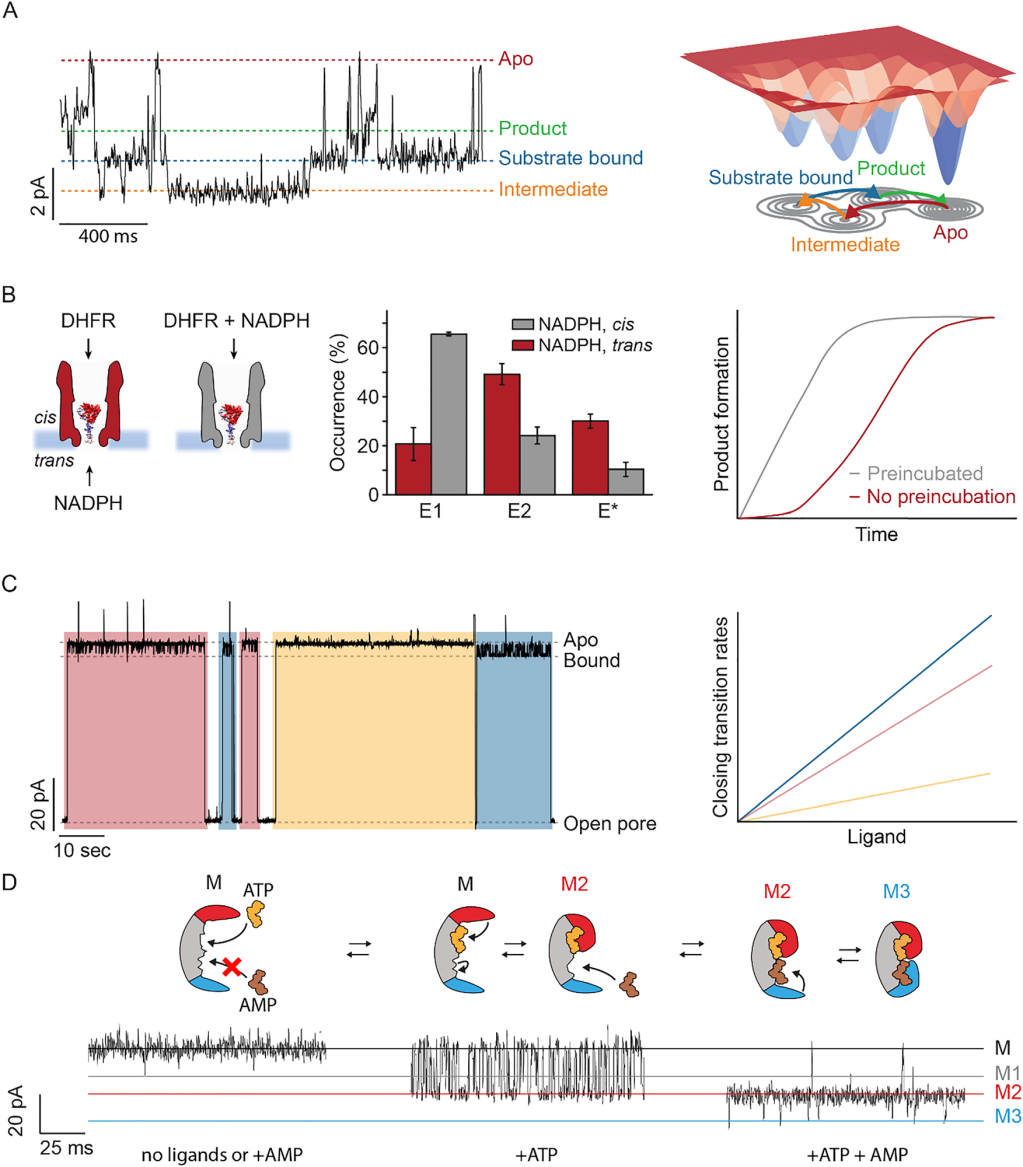
Nanopore types which are suitable for studying single-molecule enzyme dynamics. (A) Left: Representative electrophysiology trace illustrating distinct enzyme conformational states of DHFR, including the apo, intermediate, substrate bound, and product state, detected by distinct current levels. Adapted with permission from ref. 62. Copyright 2022 American Chemical Society (ACS). Right: Schematic representation of population shifts induced by ligand binding. (B) Left: Schematic representation of the reaction conditions during electrophysiology measurements of DHFR, middle: histogram of the distribution of three different DHFR conformers, with E1 being the catalytically active conformer. Right: Corresponding exemplary time-course plot of product formation. Left and middle figures adapted with permission from ref. 63. Copyright 2020 Macmillan Publishers Ltd: *Nature Chemistry*. (C) Left: Single-molecule enzyme trace of DHFR showing multiple conformational states, highlighted with blue, yellow, and red boxes. Figure adapted with permission from ref. 63. Copyright 2020 Macmillan Publishers Ltd: *Nature Chemistry*. Right: Exemplary plot of transition rates *versus* ligand concentration, revealing different ligand affinities for distinct enzyme conformations. (D) Top: Schematic representation illustrating (*endo*)allosteric regulation, where binding of the yellow substrate molecule at the allosteric site induces a conformational change in the active site, facilitating binding of the brown substrate ligand. The conformational shift enhances affinity for the allosteric regulator. The binding of ligands also induces the closing of the LID (red) and NMP (blue) domains of AK. Bottom: The corresponding trace for the different states of the enzyme. Figure adapted with permission from ref. 64. Copyright 2024 Macmillan Publishers Ltd: *Nature Communications*.

One of the most critical aspects of enzyme function is how conformational dynamics—rapid, reversible structural transitions—enable substrate binding, catalysis, and product release. These changes are tightly linked to enzyme activity and kinetics, directly influencing the speed and specificity of catalytic reactions. Among other single molecule techniques, nanopore technology enables studying global conformational dynamics of a single enzyme over long timescales. When structural information is available, this approach enables the determination of both the rates of structural changes and the connectivity between structural states, including substrate binding events (Fig. 3A). Below, we will address three examples.

Dihydrofolate reductase (DHFR) catalyzes the reaction of dihydrofolate (DHF) and NADPH to tetrahydrofolate (THF) and NADP^+^. The product THF plays an important role in the *de novo* synthesis of purines, amino acids like glutamate, and thymidylic acid. In the last decades, tens of crystal structures in the presence of substrate, cofactors, inhibitors and products revealed that the reactant binds to a closed conformation, while the product binds to an occluded conformation. DHFR could be trapped within a solid-state silicon nitride (SiN) nanopore^159^ as well as in the biological ClyA-AS nanopore.^27,62,63,144^ The limited resolution of the solid-state nanopore only allowed for the observation of the overall conformational flexibility in wild-type and mutant DHFR variants. In contrast, the biological nanopore enabled the trapping of the protein inside the nanopore and the identification of multiple enzyme conformations and their exchange rates. The enzyme populates at least four ground state conformations, called conformers. Three of these conformers have different affinity for the substrate, cofactor and products, while the fourth conformer could not bind to any ligand. The conformers were not found to interconvert over the several minutes of the trapping of the enzyme inside the nanopore. Sampling different ligands and their mixtures allowed assigning the different conformers to the closed and occluded conformations observed in the DHFR crystal structures. According to the already established catalytic mechanism,^160^ single-molecule experiments showed that substrate binding and release occurs hierarchically, with the reactive ternary complex mainly forming from the NADPH-bound closed configuration rather than from the dihydrofolate-bound occluded conformation. Only when dihydrofolate is protonated and the reaction proceeds, DHFR switches from the substrate-bound closed conformation to the product-bound occluded conformation. The product tetrahydrofolate (THF) was found to bind weakly to the closed conformation and tightly to the occluded conformation. Interestingly, the oxidized cofactor NADP^+^ was only found to bind to the closed:THF/DHF ternary complexes. Therefore, the catalytic step promotes a conformational switch to high affinity binding of THF, which in turn promotes NADP^+^ release. This switch prevents the backward reaction by the high affinity of the product for the occluded conformation. The final step involves NADPH binding, which drives the transition back to the closed conformation to facilitate the release of the product THF, underscoring the role of conformational dynamics in regulating DHFR's catalytic efficiency (Fig. 3A).^62^ Furthermore, during the reaction, a previously unobserved, long-lasting inactive conformation appeared, likely induced by the catalytic step. This suggests that DHFR occasionally adopts an alternative fold, which is not observed under nonreactive conditions.

Human cytoplasmic tyrosine Abelson (Abl) kinase is a signaling enzyme that catalyzes the transfer of a phosphate group from ATP to tyrosine residues on target proteins, regulating processes such as growth, differentiation, and survival.^161,162^ Using the ClyA nanopore, the conformational transitions of the Abl kinase domain were studied, revealing that the apo form dynamically interconverts between two major conformational states: a low-energy, lobe-open conformation and a higher-energy, lobe-closed conformation. Strikingly, the lobe-closed state further resolves into three distinct, functionally relevant sub-states that resemble substrate-binding conformations; a level of resolution of the Abl kinase's conformational landscape not previously captured by NMR or any other technique. While the substates found by NMR likely reflect the dynamics between the three different sub-states, it was not possible to resolve the two major conformational states. Quantitative analysis of transition rates and free energy differences, along with mutational and ligand-binding experiments, demonstrated that the interconversion between these states is tightly regulated by hinge flexibility and substrate engagement. Most enzymes reside in an open, inactive conformation and switch to an active conformation with a conformational closing rate of 0.09 s^−1^, while transitions between active sub-states occur at >100 s^−1^, consistent with a catalytic rate of ∼7 s^−1^.^67^

In a third example, nanopore studies with the model protein adenylate kinase (AK) provided key insights into the conformational dynamics of the enzyme. AK reversibly catalyzes ATP and AMP to two ADP molecules.^163–165^ Structural studies revealed the motions of the AMP-binding NMP and ATP-binding LID domain.^166–168^ Nanopore analysis enabled, for the first time, real-time measurement of the individual dynamic behavior of both domains. With a solid-state nanopore it was shown that the binding of the lock substrate Ap5A led to a more compact and less flexible structure compared to the free enzyme (AK-apo).^159^ It was found by means of the ClyA nanopore that during its catalytic cycle, the enzyme's LID and NMP domains move in a precise, hierarchical manner, which regulates substrate binding and catalysis (*vide infra*). The catalytic cycle begins with ATP or ADP binding, which induces the closing of the LID domain at a rate of about 1000 s^−1^, matching NMR measurements of combined NMP and LID domain motions. The LID domain can reopen at a similar rate, but when a second adenosine phosphate binds, it triggers an allosteric effect that slows the reopening of the LID domain and facilitates additional closing of the NMP domain. The NMP domain closure, the slowest conformational change at 200 s^−1^, matches AK's catalytic turnover rate (263  ±  30 s^−1^) and is likely the rate-limiting step of the reaction. This contrasts with earlier studies, which suggested that the rate-limiting step was enzyme opening. The discrepancy is likely to arise from the oversimplified two-state open–closed models used in those studies.^64^

### 3.3 Hysteresis

Hysteretic enzymes are characterized by a delayed response in activity following changes in a physical or chemical parameter (*e.g.* substrate/inhibitor concentration, pH, or temperature).^5,6^ This delay, which can range from seconds^169–171^ to hours,^172^ is primarily observed in the context of metabolic regulation (Fig. 3B, right). Hysteresis provides a form of “molecular memory” to enzymes, filtering out short-term fluctuations in substrate concentration and facilitating progression through cellular pathways, such as cell-cycle stages.^173,174^ Hysteresis in enzymes can have a variety of causes, including the replacement of a tightly bound ligand by another, oligomerization, or isomerization.^6^ However, since its introduction in 1970, it has been recognized that hysteresis is in many cases driven by the interconversion of different enzyme conformations, each with different catalytic efficiencies.^6^ To experimentally confirm the existence of these states, a variety of techniques have been employed, including structural analysis^175^ and even single-enzyme kinetics.^176,177^

Hysteresis has also been studied by nanopore technology, exemplified by DHFR from *E. coli*.^27,62,63,144,159^ DHFR exists in two catalytically different forms that interconvert at a rate of less than 0.1 s^−1^.^170,178–185^ The enzyme shows hysteresis that lasts for several seconds, and this effect is prevented by preincubation with the substrate NADPH.^171,183,184^ The single-molecule analysis revealed that DHFR exists, not in two, but in at least four ground conformers (see also Section 3.2).^63^ In a typical nanopore experiment, the protein of interest is added to only one (*cis*) side of the nanopore, whereas the substrate can be added to either side. Substrate addition from the other (*trans*) side resembles a stopped-flow experiment, where rapid mixing allows for transient states and reaction dynamics. This setup enables the recording of DHFR proteins that were either preincubated with NADPH (*cis* addition of substrate) or only encountered NADPH in the nanopore environment (*trans* addition of substrate). Preincubation with NADPH shifts the DHFR population to the catalytically more active conformer, explaining the hysteresis observed in kinetic assays (Fig. 3B).^63^ Surprisingly, the product NADP^+^ shifts the population towards a low-affinity conformer, while the addition of the transition-state mimic methotrexate, along with NADPH/NADP^+^, induces frequent exchange between the conformers. This indicates that DHFR can switch conformers along the reaction coordinate to a low-affinity conformer, facilitating substrate release. Subsequent binding of a new NADPH molecule provides the energy to revert to the high-affinity conformer. Altogether, this in-depth single-molecule analysis of a hysteretic enzyme demonstrates how hysteresis can reflect a critical enzymatic property, namely efficient substrate binding and product release.

### 3.4 The same enzyme in different forms

The presence of different conformers within a single protein pool is not unique to DHFR, but has also been described for AK.^64^ The latter enzyme exhibits allosteric domain closure upon sequential binding of the two substrates (*vide infra*). Approximately 12% of the trapped proteins display altered kinetic properties, such as a fourfold increased off-rate for ADP and a less pronounced reduction in the opening rate of one of the protein domains when the ADP concentration increases, which is indicative of lower allosteric communication (Fig. 3C). The two different AK forms are indistinguishable by their current levels, indicating a similar overall fold, but with subtle variations that impact protein function and its allosteric behavior. Similarly, the flaviviral NS2B/NS3 protease samples two open conformations, a long-lived (∼70 ms) state and short-lived (∼3 ms) state, which are also indistinguishable by their current levels but can be differentiated based on their transition rates to the closed conformation.^66^

Why should we care about these rare protein forms and conformations? There are several reasons, of which two will be addressed here. Firstly, these nanopore experiments highlight that a single chain of amino acids can fold into different forms with distinct properties and possibly different physiological functionalities.^186^ Secondly, the presence of different forms or rare conformations can be exploited in the search for new enzyme activities. For example, single-molecule kinetics experiments revealed that mutations remote from the binding pocket lead to a correlated increase in the diversity in functional states and promiscuity.^187^ Promiscuity is a valuable trait in evolution, enabling the development of new enzyme functions, and is also an indispensable property in the field of biocatalysts. Even minimal promiscuous activity towards a desired reaction can serve as a foundation for engineering enzymes with high activity in biotechnological applications.^188–190^ The visualization and quantification of rare protein forms with nanopores contribute to the thermodynamic framework that rationalizes how mutations can alter enzyme function through reshaping the enzyme conformational distribution, thus enriching catalytically productive states and reducing non-productive ones.

### 3.5 (*endo*-)Allostery

Allostery in enzymes refers to a process in which the binding of a ligand (or effector molecule) to one site on the enzyme induces a change that affects the enzyme's activity at a distant site, typically the active site.^2,3,191,192^ This allows enzymes to adjust their activity in response to changes in the cellular environment, providing a mechanism for precise metabolic regulation.

Allostery is a dynamic process driven by shifts in a protein's thermodynamic ensemble of conformational states. Modern perspectives describe it as a statistical ensemble of states, where proteins exist in a mixture of conformations that change in response to external perturbations.^193–196^ Ligand binding, for example, remodels the protein's energy landscape, altering the stability of various conformations and influencing function.^197,198^ This remodeling can shift the equilibrium toward activation or repression, or adjust the coupling between functional domains. The coupling, either positive or negative, affects how ligand binding influences other sites. Dynamic allostery extends beyond structural changes, emphasizing the role of protein flexibility and conformational entropy. In many cases, changes in protein dynamics, such as side-chain flexibility^199,200^ or localized unfolding,^201,202^ can drive allosteric transitions without significant structural shifts. This is evident in proteins with intrinsically disordered regions. For example, intrinsically disordered proteins mediate allosteric effects through their inherent flexibility, allowing adaptation of their conformational ensemble in response to binding events.^203–205^ Allostery in nanopore experiments manifests as the emergence of a new conformational state or as alterations in the dynamics of existing states (Fig. 3D).^64,206,207^

Using the MspA nanopore, the allosteric behavior of the non-enzymatic protein calmodulin (CaM) was addressed by distinguishing its conformational changes in response to Ca^2+^ binding. In the absence of Ca^2+^, calmodulin is in a “closed” or inactive state, and it does not bind effectively to the M13 peptide or other target proteins. The nanopore detected three distinct states of CaM: Ca^2+^-free, Ca^2+^-bound, and M13 peptide-bound, illustrating how calcium ions induce a conformational shift that enables the M13 peptide binding. The sensitivity of MspA also allows detection of subtle structural changes, such as those caused by a single amino acid mutation (D129G), which affects Ca^2+^ binding. Additionally, various other ions were tested for their ability to induce the conformational change, showing that ions, such as Pb^2+^, Ca^2+^ and Sr^2+^, effectively trigger the structural shift.^206^

A ligand–receptor-anchored glass nanopore system was introduced to probe the dynamic binding pathways of multivalent protein–protein interactions. Using soluble angiotensin converting enzyme 2 (sACE2) as a receptor, the system monitored real-time interactions with the trimeric spike proteins from the Omicron, Delta, and WT SARS-CoV-2 variants which were trapped inside the glass nanotube. The results revealed that Omicron's spike protein exhibits strong, cooperative binding across all three S1 monomers, following a concentration-dependent, multistep pathway. Initial sACE2 binding enhances subsequent interactions—an allosteric effect not observed in Delta and WT variants, which mainly bind to one or two monomers. Although the first binding step of Omicron is weaker than Delta's, the second and third steps show significantly higher affinity. These findings highlight an allosteric mechanism in the spike-sACE2 complex, explaining Omicron's increased infectivity and offering new insights into multivalent protein interactions.^207^

Finally, the LID domain of AK does not fully close when ATP alone is bound. However, upon adding AMP, which binds the NMP domain, the LID domain fully closes. This conformational change suggests a cooperative interaction, with AMP driving the complete closure of the LID domain. To describe the intriguing process, where the reactants themselves, ATP and AMP, mediate the allosteric effect within the same reactive site, the term “*endo*-allostery” was introduced. This mechanism differs from cooperativity, in which both reactants bind to the same site, highlighting the unique role of AMP in driving AK's conformational change. These structural changes are not incidental but are essential for catalysis. The binding of AMP and ATP initiates a sequence of movements that align AK's catalytic domains, ensuring that substrate binding follows a defined order and minimizing wasteful ATP hydrolysis. In this way, allostery is directly harnessed to regulate reaction progression. Thus, *endo*-allostery in AK is not just a regulatory feature, but it is a core element of how the enzyme catalyzes its reaction efficiently and in a precise order.^64^

## 4. Outlook

Currently, the contribution of nanopore experiments to single-molecule enzymology, by trapping them within the pores, is limited to a small number of enzymes that have undergone in-depth analysis. Additionally, some larger enzymes (>100 kDa) have been demonstrated to be compatible with nanopore analysis (Table 2). In the final section of this review, we will discuss the current challenges facing the nanopore field and explore potential strategies for unlocking the full potential of nanopores in single-molecule enzymology.

**Table 2:** Summary of enzymes whose dynamics have been analyzed within a nanopore

Enzyme	Size (kDa)	Organism	Nanopore type		Trapping time	Ref.
Dihydrofolate reductase (DHFR)	18	*E. coli*	Biological	ClyA	Minutes	27,62,63,144
Abelson 1 kinase (Abl kinase)	33	*Homo sapiens*	Biological	ClyA	Seconds	67
NS2B/NS3 protease	28	West Nile virus	Biological	ClyA	Seconds	66
Adenylate kinase (AK)	24	*E. coli*	Biological	ClyA	Seconds	64
Alkylation B (AlkB)	25	*E. coli*	Biological	ClyA	Minutes	27
Glucose oxidase	160	*Aspergillus niger*	Solid-state	Silicon-nitride	Minutes	65
Heat shock protein 90 (Hsp90)	163	*Saccharomyces cerevisiae*	Solid-state	Silicon-nitride; NEOtrap	Hours	120

### 4.1 Biological nanopores

Most single-molecule enzymology studies using nanopores have been performed with the biological nanopore cytolysin A (ClyA) from either *E. coli* or *Salmonella typhi*. Its geometry of stacked small and large cylinders allows for efficient protein capture in a confined space. Directed evolution has improved ClyA from *S. typhi* in terms of solubility and stability.^87^ However, maximizing the capabilities of biological nanopores requires exploration of alternative approaches.

Firstly, ClyA forms pores of different oligomeric sizes which have an inner diameter of 5.5–6.5 nm on the enzyme entry side and 3.3–4.2 nm on the transmembrane side. This geometry limits the enzyme size range to enzymes small enough to enter the pore but large enough to prevent rapid translocation (typically 15–45 kDa proteins). To accommodate enzymes outside this size range, alternative nanopores are needed. Smaller enzymes can be captured using nanopores like *Mycobacterium smegmatis* MspA^29,208,209^ or φ29 phage DNA packaging motor (Fig. 2).^210,211^ Conversely, larger enzymes can be trapped within *Pleurotus ostreatus* PlyAB,^212,213^*Yersinia enterocolitica* YaxAB,^37,88,214^ or *Xenorhabdus nematophila* XaxAB (Fig. 2).^215^ PlyAB, for instance, has a cylindrical structure with a 7.2 nm and 10.5 nm entry side, and a 5.5 nm inner constriction. Unlike ClyA and PlyAB, YaxAB has a conical shape, making it suitable for capturing a wide range of protein sizes (12–125 kDa). For even bigger enzymes, the YaxAB homolog XaxAB can be employed, as it has a similar geometry while forming higher oligomeric pores. The modular nature of biological components enables the creation of customizable nanopore systems, such as hetero-oligomeric assemblies.^216^ Similarly, the 900 kDa multiprotein complex, used for protein sequencing, exemplifies how diverse biological components can be combined in one pore.^45^

Secondly, a limitation of (most) biological nanopores is their inherent negative charge, like many biological macromolecules.^217,218^ This leads to the electro-osmotic and electrophoretic force acting in opposite directions on negatively charged enzymes, reducing capturing frequency and trapping time. As described in Section 2, the issue can be solved by adding a positively charged electrophoretic tag to the enzyme of interest.^64,67,144^ Alternatively, the constriction of the pore can be mutated to contain positive charges to reverse the electro-osmotic flow.^37,86,219^ The highly charged nanopore lumen can also cause electrostatic repulsion, preventing stable enzyme trapping in a single energy minimum. Consequently, the current fluctuations may reflect enzyme repositioning inside the pore rather than conformational dynamics.^64,66,87,149^ Mutations to both the enzyme and the nanopore can facilitate stable enzyme trapping, and engineering less charged nanopores is a promising but underexplored area that could significantly improve the trapping stability.

Thirdly, compared to solid-state nanopores, lipid membranes incorporating biological nanopores have lower stability and durability. Although a stable pore can survive for several hours, the lipid membrane is prone to breaking and, in some cases, allows the nanopore to detach, particularly during mixing steps or when membrane-active compounds are present in solution. This hinders the sampling of membrane-bound enzymes or enzymes with lipid ligands, such as phospholipases. In the case of membrane-bound enzymes, these agents can even deliver the membrane protein to the lipid membrane or destabilize it altogether.^220–222^ Additionally, nanopores often exhibit a preference for specific bilayer compositions for insertion.^40,223–225^ The stability of lipid bilayers can be greatly improved by using a mixture of lipids and block copolymers.^226^ Alternatively, future efforts should focus on developing a hybrid nanopore, combining the durability of solid-state nanopores with the sensitivity of biological nanopores. The concept of a hybrid pore was first demonstrated by embedding a biological nanopore within a solid-state nanopore, which was stable for several days but had high leakage currents due to an imperfect seal.^227^ While recent advancements in creating these hybrid pores are promising, they are not yet used in single-molecule enzyme research.^228–231^

### 4.2 Solid-state nanopores

Solid-state nanopores offer several advantages over biological nanopores, including greater stability, the ability to be manufactured in arrays to increase data output, and their availability in a wide range of sizes.^78^ Nonetheless, their use with regard to single-molecule enzymology has been limited. The geometry of the pores prevents stable enzyme trapping, restricting dynamic information to be extracted from short translocation events.^159,232–234^ Alternatively, there are ways to trap enzymes for longer periods of time, such as slowing down protein translocation to milliseconds by Ni^2+^-NTA/His-tag,^235^ streptavidin/biotin,^100^ or NHS/protein interactions^65,236^ within coated nanopores. For longer observation times, ranging from seconds to minutes, a peptide linked to monovalent streptavidin *via* a biotin tag has been used to block the nanopore without translocation (Fig. 2).^237^ However, this approach inherently limits the maximal size of the studied protein and introduces the need for protein labeling. As described in Section 2, most promising has been the recent development of the NEOtrap, which has been used to reveal different conformational states in the ∼160 kDa yeast chaperone Hsp90 dimer (Fig. 2).^120,148,238^

### 4.3 Future prospects

Nanopore technology provided exquisite and detailed information on the structural dynamics of small enzymes, offering novel explanations for longstanding enzymology concepts. The technology is well suited for uncovering mechanistic insights in enzymes that display allostery, hysteresis, or kinetic cooperativity. The advent of larger biological nanopores and the development of the NEOtrap expand the potential for studying larger and multidomain enzymes or even oligomeric enzyme complexes. While sub-nanometer conformational changes have been detected in some small enzymes, achieving comparable resolution for large enzymes remains uncertain. The power of nanopores lies in the ability to report on global structural changes; however, correlating current levels with specific conformations becomes increasingly challenging with increasing enzyme size and complexity. Molecular dynamics (MD) simulations provide a valuable solution by predicting protein conformational behavior within the nanopore and the corresponding current changes.^37,66,144^ Furthermore, machine learning approaches have been successfully employed to automatically decode DNA/RNA/peptide sequences from current traces, and to detect proteins and protein–substrate interactions within pores.^239–242^ These methods could similarly be applied to interpret complex current traces in the context of enzyme dynamics.

None of the current methodologies can report on the full spectrum of enzyme dynamics. Therefore, an advantage lies in the combination of different techniques in a single measuring instrument, as is done by combining single-molecule optical tweezers with single-molecule FRET spectroscopy.^26,243^ Likewise, solid-state nanopore electrical recordings have been combined with fluorescence microscopy, to simultaneously acquire electrical and optical information.^244–248^ We envision that integrating nanopores with other single-molecule techniques will enable a more comprehensive understanding of enzyme dynamics.

Altogether, the rapid advancement of diverse nanopore systems, together with pioneering studies proving their capability to uncover detailed single-molecule enzymatic kinetics, holds great promise for future research. The time is ripe for a wider application of single-molecule nanopore enzymology.

## Author contributions

NG and MN wrote the review article. GM supervised the process. All authors verified the manuscript.

## Conflicts of interest

GM is founder, director, and shareholder of Portal Biotech Limited, a company engaged in the development of nanopore technologies. NG and MN declare no competing financial interests.

## Data Availability

No primary research results, software or code have been included and no new data were generated or analysed as part of this review.

## References

[cit1] Kühne W. (1877). Über das Verhalten Verschiedener organisirter und sog. ungeformter Fermente. Neue Folge Heidelberg.

[cit2] Monod J., Jacob F. (1961). General Conclusions: Teleonomic Mechanisms in Cellular Metabolism, Growth, and Differentiation. Cold Spring Harbor Symp. Quant. Biol..

[cit3] Changeux J. P. (1961). The Feedback Control Mechanism of Biosynthetic L-Threonine Deaminase by L-Isoleucine. Cold Spring Harbor Symp. Quant. Biol..

[cit4] Monod J., Wyman J., Changeux J. P. (1965). On the nature of allosteric transitions: A plausible model. J. Mol. Biol..

[cit5] Neet K. E. and Robert Ainslie G., *[8] Hysteretic enzymes*, 1980, pp. 192–226 10.1016/s0076-6879(80)64010-5 7374453

[cit6] Frieden C. (1970). Kinetic Aspects of Regulation of Metabolic Processes. J. Biol. Chem..

[cit7] Abramson J., Adler J., Dunger J., Evans R., Green T., Pritzel A. (2024). *et al.*, Accurate structure prediction of biomolecular interactions with AlphaFold 3. Nature.

[cit8] Hilvert D. (2000). Critical Analysis of Antibody Catalysis. Annu. Rev. Biochem..

[cit9] Hekkelman M. L., de Vries I., Joosten R. P., Perrakis A. (2023). AlphaFill: enriching AlphaFold models with ligands and cofactors. Nat. Methods.

[cit10] Nie Y., Wang S., Xu Y., Luo S., Zhao Y. L., Xiao R. (2018). *et al.*, Enzyme Engineering Based on X-ray Structures and Kinetic Profiling of Substrate Libraries: Alcohol Dehydrogenases for Stereospecific Synthesis of a Broad Range of Chiral Alcohols. ACS Catal..

[cit11] Kaczmarski J. A., Mahawaththa M. C., Feintuch A., Clifton B. E., Adams L. A., Goldfarb D. (2020). *et al.*, Altered conformational sampling along an evolutionary trajectory changes the catalytic activity of an enzyme. Nat. Commun..

[cit12] Campbell E. C., Correy G. J., Mabbitt P. D., Buckle A. M., Tokuriki N., Jackson C. J. (2018). Laboratory evolution of protein conformational dynamics. Curr. Opin. Struct. Biol..

[cit13] Campbell E., Kaltenbach M., Correy G. J., Carr P. D., Porebski B. T., Livingstone E. K. (2016). *et al.*, The role of protein dynamics in the evolution of new enzyme function. Nat. Chem. Biol..

[cit14] Casilli F., Canyelles-Niño M., Roelfes G., Alonso-Cotchico L. (2024). Computation-guided engineering of distal mutations in an artificial enzyme. Faraday Discuss..

[cit15] Henzler-Wildman K., Kern D. (2007). Dynamic personalities of proteins. Nature.

[cit16] Bar-Even A., Milo R., Noor E., Tawfik D. S. (2015). The Moderately Efficient Enzyme: Futile Encounters and Enzyme Floppiness. Biochemistry.

[cit17] Kernohan J. C. (1965). The pH-activity curve of bovine carbonic anhydrase and its relationship to the inhibition of the enzyme by anions. Biochim. Biophys. Acta, Enzymol. Biol. Oxid..

[cit18] Khalifah R. G. (1971). The Carbon Dioxide Hydration Activity of Carbonic Anhydrase. J. Biol. Chem..

[cit19] Ellis R. J. (1979). The most abundant protein in the world. Trends Biochem. Sci..

[cit20] Jeschke G. (2012). DEER Distance Measurements on Proteins. Annu. Rev. Phys. Chem..

[cit21] Zheng J., Strutzenberg T., Pascal B. D., Griffin P. R. (2019). Protein dynamics and conformational changes explored by hydrogen/deuterium exchange mass spectrometry. Curr. Opin. Struct. Biol..

[cit22] Napoli F., Becker L. M., Schanda P. (2023). Protein dynamics detected by magic-angle spinning relaxation dispersion NMR. Curr. Opin. Struct. Biol..

[cit23] Schanda P., Haran G. (2024). NMR and Single-Molecule FRET Insights into Fast Protein Motions and Their Relation to Function. Annu. Rev. Biophys..

[cit24] Lerner E., Cordes T., Ingargiola A., Alhadid Y., Chung S., Michalet X. (2018). *et al.*, Toward dynamic structural biology: Two decades of single-molecule Förster resonance energy transfer. Science.

[cit25] Ando T. (2019). High-speed atomic force microscopy. Curr. Opin. Chem. Biol..

[cit26] Bustamante C. J., Chemla Y. R., Liu S., Wang M. D. (2021). Optical tweezers in single-molecule biophysics. Nat. Rev. Methods Primers.

[cit27] Soskine M., Biesemans A., Maglia G. (2015). Single-Molecule Analyte Recognition with ClyA Nanopores Equipped with Internal Protein Adaptors. J. Am. Chem. Soc..

[cit28] Maglia G., Heron A. J., Stoddart D., Japrung D., Bayley H. (2010). Analysis of single nucleic acid molecules with protein nanopores. Methods Enzymol..

[cit29] Manrao E. A., Derrington I. M., Laszlo A. H., Langford K. W., Hopper M. K., Gillgren N. (2012). *et al.*, Reading DNA at single-nucleotide resolution with a mutant MspA nanopore and phi29 DNA polymerase. Nat. Biotechnol..

[cit30] Clarke J., Wu H. C., Jayasinghe L., Patel A., Reid S., Bayley H. (2009). Continuous base identification for single-molecule nanopore DNA sequencing. Nat. Nanotechnol..

[cit31] Wang Y. Y., Zhao Y., Bollas A., Wang Y. Y., Au K. F. (2021). Nanopore sequencing technology, bioinformatics and applications. Nat. Biotechnol..

[cit32] Branton D., Deamer D. W., Marziali A., Bayley H., Benner S. A., Butler T. (2008). *et al.*, The potential and challenges of nanopore sequencing. Nat. Biotechnol..

[cit33] Deamer D., Akeson M., Branton D. (2016). Three decades of nanopore sequencing. Nat. Biotechnol..

[cit34] Ying Y. L., Hu Z. L., Zhang S., Qing Y., Fragasso A., Maglia G. (2022). *et al.*, Nanopore-based technologies beyond DNA sequencing. Nat. Nanotechnol..

[cit35] Wloka C. , Galenkamp N. S., van der Heide N. J., Lucas F. L. R. R. and Maglia G., Chapter Nineteen – Strategies for enzymological studies and measurements of biological molecules with the cytolysin A nanopore, in Pore-Forming Toxins, ed. A. P. Heuck, Academic Press, 2021, pp. 567–585 10.1016/bs.mie.2021.01.007 33712200

[cit36] Zhang X., Galenkamp N. S., van der Heide N. J., Moreno J., Maglia G., Kjems J. (2023). Specific Detection of Proteins by a Nanobody-Functionalized Nanopore Sensor. ACS Nano.

[cit37] Straathof S., Di Muccio G., Yelleswarapu M., Alzate Banguero M., Wloka C., van der Heide N. J. (2023). *et al.*, Protein Sizing with 15 nm Conical Biological Nanopore YaxAB. ACS Nano.

[cit38] Zhang M., Tang C., Wang Z., Chen S., Zhang D., Li K. (2024). *et al.*, Real-time detection of 20 amino acids and discrimination of pathologically relevant peptides with functionalized nanopore. Nat. Methods.

[cit39] Galenkamp N. S., Soskine M., Hermans J., Wloka C., Maglia G. (2018). Direct electrical quantification of glucose and asparagine from bodily fluids using nanopores. Nat. Commun..

[cit40] Thakur A. K., Movileanu L. (2019). Real-time measurement of protein–protein interactions at single-molecule resolution using a biological nanopore. Nat. Biotechnol..

[cit41] Lu C., Bonini A., Viel J. H., Maglia G. (2025). Toward single-molecule protein sequencing using nanopores. Nat. Biotechnol..

[cit42] Motone K., Kontogiorgos-Heintz D., Wee J., Kurihara K., Yang S., Roote G. (2024). *et al.*, Multi-pass, single-molecule nanopore reading of long protein strands. Nature.

[cit43] Yan S., Zhang J., Wang Y., Guo W., Zhang S., Liu Y. (2021). *et al.*, Single Molecule Ratcheting Motion of Peptides in a Mycobacterium smegmatis Porin A (MspA) Nanopore. Nano Lett..

[cit44] Brinkerhoff H., Kang A. S. W., Liu J., Aksimentiev A., Dekker C. (2021). Multiple rereads of single proteins at single–amino acid resolution using nanopores. Science.

[cit45] Zhang S., Huang G., Versloot R. C. A., Bruininks B. M. H., de Souza P. C. T., Marrink S. J. (2021). *et al.*, Bottom-up fabrication of a proteasome–nanopore that unravels and processes single proteins. Nat. Chem..

[cit46] Kwak D. K., Kim J. S., Lee M. K., Ryu K. S., Chi S. W. (2020). Probing the Neuraminidase Activity of Influenza Virus Using a Cytolysin A Protein Nanopore. Anal. Chem..

[cit47] Zhao Q., de Zoysa R. S. S., Wang D., Jayawardhana D. A., Guan X. (2009). Real-time monitoring of peptide cleavage using a nanopore probe. J. Am. Chem. Soc..

[cit48] Majd S., Yusko E. C., MacBriar A. D., Yang J., Mayer M. (2009). Gramicidin Pores Report the Activity of Membrane-Active Enzymes. J. Am. Chem. Soc..

[cit49] Rauf S., Zhang L., Ali A., Ahmad J., Liu Y., Li J. (2017). Nanopore-Based, Label-Free, and Real-Time Monitoring Assay for DNA Methyltransferase Activity and Inhibition. Anal. Chem..

[cit50] Meng F. N., Ying Y. L., Yang J., Long Y. T. (2019). A Wild-Type Nanopore Sensor for Protein Kinase Activity. Anal. Chem..

[cit51] Jiang J., Li M. Y., Wu X. Y., Ying Y. L., Han H. X., Long Y. T. (2023). Protein nanopore reveals the renin–angiotensin system crosstalk with single-amino-acid resolution. Nat. Chem..

[cit52] Wloka C., Van Meervelt V., Van Gelder D., Danda N., Jager N., Williams C. P. (2017). *et al.*, Label-Free and Real-Time Detection of Protein Ubiquitination with a Biological Nanopore. ACS Nano.

[cit53] Wang F., Zahid O. K., Swain B. E., Parsonage D., Hollis T., Harvey S. (2017). *et al.*, Solid-State Nanopore Analysis of Diverse DNA Base Modifications Using a Modular Enzymatic Labeling Process. Nano Lett..

[cit54] Cherf G. M., Lieberman K. R., Rashid H., Lam C. E., Karplus K., Akeson M. (2012). Automated forward and reverse ratcheting of DNA in a nanopore at 5-Å precision. Nat. Biotechnol..

[cit55] Lieberman K. R., Cherf G. M., Doody M. J., Olasagasti F., Kolodji Y., Akeson M. (2010). Processive Replication of Single DNA Molecules in a Nanopore Catalyzed by phi29 DNA Polymerase. J. Am. Chem. Soc..

[cit56] Dahl J. M., Lieberman K. R., Wang H. (2016). Modulation of DNA Polymerase Noncovalent Kinetic Transitions by Divalent Cations. J. Biol. Chem..

[cit57] Craig J. M., Laszlo A. H., Nova I. C., Brinkerhoff H., Noakes M. T., Baker K. S. (2019). *et al.*, Determining the effects of DNA sequence on Hel308 helicase translocation along single-stranded DNA using nanopore tweezers. Nucleic Acids Res..

[cit58] Nivala J., Marks D. B., Akeson M. (2013). Unfoldase-mediated protein translocation through an α-hemolysin nanopore. Nat. Biotechnol..

[cit59] Ho C. W., Van Meervelt V., Tsai K. C., De Temmerman P. J., Mast J., Maglia G. (2015). Engineering a nanopore with co-chaperonin function. Sci. Adv..

[cit60] Harrington L., Alexander L. T., Knapp S., Bayley H. (2019). Single-Molecule Protein Phosphorylation and Dephosphorylation by Nanopore Enzymology. ACS Nano.

[cit61] Harrington L., Alexander L. T., Knapp S., Bayley H. (2015). Pim Kinase Inhibitors Evaluated with a Single-Molecule Engineered Nanopore Sensor. Angew. Chem., Int. Ed..

[cit62] Galenkamp N. S., Maglia G. (2022). Single-Molecule Sampling of Dihydrofolate Reductase Shows Kinetic Pauses and an Endosteric Effect Linked to Catalysis. ACS Catal..

[cit63] Galenkamp N. S., Biesemans A., Maglia G. (2020). Directional conformer exchange in dihydrofolate reductase revealed by single-molecule nanopore recordings. Nat. Chem..

[cit64] Galenkamp N. S., Zernia S., Van Oppen Y. B., van den Noort M., Milias-Argeitis A., Maglia G. (2024). Allostery can convert binding free energies into concerted domain motions in enzymes. Nat. Commun..

[cit65] Yu R. J., Li Q., Liu S. C., Ma H., Ying Y. L., Long Y. T. (2023). Simultaneous observation of the spatial and temporal dynamics of single enzymatic catalysis using a solid-state nanopore. Nanoscale.

[cit66] Shorkey S. A., Zhang Y., Sharp J., Clingman S., Nguyen L., Chen J. (2025). *et al.*, Tracking flaviviral protease conformational dynamics by tuning single-molecule nanopore tweezers. Biophys. J..

[cit67] Li F., Fahie M. A., Gilliam K. M., Pham R., Chen M. (2022). Mapping the conformational energy landscape of Abl kinase using ClyA nanopore tweezers. Nat. Commun..

[cit68] Soskine M., Biesemans A., Moeyaert B., Cheley S., Bayley H., Maglia G. (2012). An engineered ClyA nanopore detects folded target proteins by selective external association and pore entry. Nano Lett..

[cit69] Willems K., Van Meervelt V., Wloka C., Maglia G. (2017). Single-molecule nanopore enzymology. Philos. Trans. R. Soc. London, Ser. B.

[cit70] Sheng Y., Zhang S., Liu L., Wu H. (2020). Measuring Enzymatic Activities with Nanopores. ChemBioChem.

[cit71] Schmid S., Dekker C. (2021). Nanopores: a versatile tool to study protein dynamics. Essays Biochem..

[cit72] Haque F., Li J., Wu H. C., Liang X. J., Guo P. (2013). Solid-state and biological nanopore for real-time sensing of single chemical and sequencing of DNA. Nano Today.

[cit73] Waduge P., Hu R., Bandarkar P., Yamazaki H., Cressiot B., Zhao Q. (2017). *et al.*, Nanopore-Based Measurements of Protein Size, Fluctuations, and Conformational Changes. ACS Nano.

[cit74] Bayley H., Cremer P. S. (2001). Stochastic sensors inspired by biology. Nature.

[cit75] Wu D., Bi S., Zhang L., Yang J. (2014). Single-Molecule Study of Proteins by Biological Nanopore Sensors. Sensors.

[cit76] Li J., Stein D., McMullan C., Branton D., Aziz M. J., Golovchenko J. A. (2001). Ion-beam sculpting at nanometre length scales. Nature.

[cit77] Deng T., Li M., Wang Y., Liu Z. (2015). Development of solid-state nanopore fabrication technologies. Sci. Bull..

[cit78] Xue L., Yamazaki H., Ren R., Wanunu M., Ivanov A. P., Edel J. B. (2020). Solid-state nanopore sensors. Nat. Rev. Mater..

[cit79] Guan X., Li H., Chen L., Qi G., Jin Y. (2023). Glass Capillary-Based Nanopores for Single Molecule/Single Cell Detection. ACS Sens..

[cit80] Wei R., Martin T. G., Rant U., Dietz H. (2012). DNA Origami Gatekeepers for Solid-State Nanopores. Angew. Chem., Int. Ed..

[cit81] Bell N. A. W., Engst Christian R., Ablay M., Divitini G., Ducati C., Liedl T. (2012). *et al.*, DNA Origami Nanopores. Nano Lett..

[cit82] Gu L. Q., Braha O., Conlan S., Cheley S., Bayley H. (1999). Stochastic sensing of organic analytes by a pore-forming protein containing a molecular adapter. Nature.

[cit83] Song L., Hobaugh M. R., Shustak C., Cheley S., Bayley H., Gouaux J. E. (1996). Structure of Staphylococcal alpha-Hemolysin, a Heptameric Transmembrane Pore. Science.

[cit84] Kasianowicz J. J., Brandin E., Branton D., Deamer D. W. (1996). Characterization of individual polynucleotide molecules using a membrane channel. Proc. Natl. Acad. Sci. U. S. A..

[cit85] Huang G., Voet A., Maglia G. (2019). FraC nanopores with adjustable diameter identify the mass of opposite-charge peptides with 44 Dalton resolution. Nat. Commun..

[cit86] Wloka C., Mutter N. L., Soskine M., Maglia G. (2016). Alpha-Helical Fragaceatoxin C Nanopore Engineered for Double-Stranded and Single-Stranded Nucleic Acid Analysis. Angew. Chem., Int. Ed..

[cit87] Soskine M., Biesemans A., De Maeyer M., Maglia G. (2013). Tuning the Size and Properties of ClyA Nanopores Assisted by Directed Evolution. J. Am. Chem. Soc..

[cit88] Jeong K. B., Ryu M., Kim J. S., Kim M., Yoo J., Chung M. (2023). *et al.*, Single-molecule fingerprinting of protein-drug interaction using a funneled biological nanopore. Nat. Commun..

[cit89] Cao C., Ying Y. L., Hu Z. L., Liao D. F., Tian H., Long Y. T. (2016). Discrimination of oligonucleotides of different lengths with a wild-type aerolysin nanopore. Nat. Nanotechnol..

[cit90] Iacovache I., Paumard P., Scheib H., Lesieur C., Sakai N., Matile S. (2006). *et al.*, A rivet model for channel formation by aerolysin-like pore-forming toxins. EMBO J..

[cit91] Butler T. Z., Pavlenok M., Derrington I. M., Niederweis M., Gundlach J. H. (2008). Single-molecule DNA detection with an engineered MspA protein nanopore. Proc. Natl. Acad. Sci. U. S. A..

[cit92] Wang J., Prajapati J. D., Gao F., Ying Y. L., Kleinekathöfer U., Winterhalter M. (2022). *et al.*, Identification of Single Amino Acid Chiral and Positional Isomers Using an Electrostatically Asymmetric Nanopore. J. Am. Chem. Soc..

[cit93] Fahie M. A., Yang B., Mullis M., Holden M. A., Chen M. (2015). Selective Detection of Protein Homologues in Serum Using an OmpG Nanopore. Anal. Chem..

[cit94] Ahmad M., Ha J. H., Mayse L. A., Presti M. F., Wolfe A. J., Moody K. J. (2023). *et al.*, A generalizable nanopore sensor for highly specific protein detection at single-molecule precision. Nat. Commun..

[cit95] Yang J., Wang K., Zhang S., Zheng X., Cui T., Yang X. (2023). *et al.*, Site-Specific Introduction of Bioorthogonal Handles to Nanopores by Genetic Code Expansion. Angew. Chem., Int. Ed..

[cit96] Varongchayakul N., Song J., Meller A., Grinstaff M. W. (2018). Single-molecule protein sensing in a nanopore: a tutorial. Chem. Soc. Rev..

[cit97] Dekker C. (2007). Solid-state nanopores. Nat. Nanotechnol..

[cit98] Movileanu L. , Single-molecule detection of proteins using nanopores, Frontiers in Sensing, Springer Vienna, Vienna, 2012, pp. 363–381

[cit99] Awasthi S., Sriboonpeng P., Ying C., Houghtaling J., Shorubalko I., Marion S. (2020). *et al.*, Polymer Coatings to Minimize Protein Adsorption in Solid-State Nanopores. Small Methods.

[cit100] Yusko E. C., Johnson J. M., Majd S., Prangkio P., Rollings R. C., Li J. (2011). *et al.*, Controlling protein translocation through nanopores with bio-inspired fluid walls. Nat. Nanotechnol..

[cit101] Awasthi S., Ying C., Li J., Mayer M. (2023). Simultaneous Determination of the Size and Shape of Single α-Synuclein Oligomers in Solution. ACS Nano.

[cit102] Berger C., Weber-Bornhauser S., Eggenberger J., Hanes J., Plückthun A., Bosshard H. R. (1999). Antigen recognition by conformational selection. FEBS Lett..

[cit103] Eggenberger O. M., Ying C., Mayer M. (2019). Surface coatings for solid-state nanopores. Nanoscale.

[cit104] Eggenberger O. M., Leriche G., Koyanagi T., Ying C., Houghtaling J., Schroeder T. B. H. (2019). *et al.*, Fluid surface coatings for solid-state nanopores: comparison of phospholipid bilayers and archaea-inspired lipid monolayers. Nanotechnology.

[cit105] Storm A. J., Chen J. H., Ling X. S., Zandbergen H. W., Dekker C. (2003). Fabrication of solid-state nanopores with single-nanometre precision. Nat. Mater..

[cit106] Siwy Z. S. (2006). Ion-Current Rectification in Nanopores and Nanotubes with Broken Symmetry. Adv. Funct. Mater..

[cit107] Kwok H., Briggs K., Tabard-Cossa V. (2014). Nanopore Fabrication by Controlled Dielectric Breakdown. PLoS One.

[cit108] Goto Y., Matsui K., Yanagi I., Takeda K. I. (2019). Silicon nitride nanopore created by dielectric breakdown with a divalent cation: Deceleration of translocation speed and identification of single nucleotides. Nanoscale.

[cit109] Bandara Y. M. N. D. Y., Karawdeniya B. I., Hagan J. T., Chevalier R. B., Dwyer J. R. (2019). Chemically Functionalizing Controlled Dielectric Breakdown Silicon Nitride Nanopores by Direct Photohydrosilylation. ACS Appl. Mater. Interfaces.

[cit110] Henriquez R. R., Ito T., Sun L., Crooks R. M. (2004). The resurgence of Coulter counting for analyzing nanoscale objects. Analyst.

[cit111] Qiu Y., Vlassiouk I., Chen Y., Siwy Z. S. (2016). Direction Dependence of Resistive-Pulse Amplitude in Conically Shaped Mesopores. Anal. Chem..

[cit112] Li W., Bell N. A. W., Hernández-Ainsa S., Thacker V. V., Thackray A. M., Bujdoso R. (2013). *et al.*, Single Protein Molecule Detection by Glass Nanopores. ACS Nano.

[cit113] Li G. X., Zhang Z. X., Lin X. Q. (2010). Fabrication of Glass Nanopore Electrodes for Single-molecule Detection of β-Cyclodextrin. Chin. J. Anal. Chem..

[cit114] Gao C., Ding S., Tan Q., Gu L. Q. (2009). Method of Creating a Nanopore-Terminated Probe for Single-Molecule Enantiomer Discrimination. Anal. Chem..

[cit115] Sha J., Si W., Xu W., Zou Y., Chen Y. (2015). Glass capillary nanopore for single molecule detection. Sci. China: Technol. Sci..

[cit116] Nguyen T. D., Song M. S., Ly N. H., Lee S. Y., Joo S. (2019). Nanostars on Nanopipette Tips: A Raman Probe for Quantifying Oxygen Levels in Hypoxic Single Cells and Tumours. Angew. Chem., Int. Ed..

[cit117] Lu S. M., Long Y. T. (2019). Confined Nanopipette-A new microfluidic approach for single cell analysis. TrAC, Trends Anal. Chem..

[cit118] Fragasso A., Schmid S., Dekker C. (2020). Comparing Current Noise in Biological and Solid-State Nanopores. ACS Nano.

[cit119] Tabard-Cossa V., Trivedi D., Wiggin M., Jetha N. N., Marziali A. (2007). Noise analysis and reduction in solid-state nanopores. Nanotechnology.

[cit120] Schmid S., Stömmer P., Dietz H., Dekker C. (2021). Nanopore electro-osmotic trap for the label-free study of single proteins and their conformations. Nat. Nanotechnol..

[cit121] Hernández-Ainsa S., Keyser U. F. (2014). DNA origami nanopores: developments, challenges and perspectives. Nanoscale.

[cit122] Bell N. A. W., Keyser U. F. (2014). Nanopores formed by DNA origami: A review. FEBS Lett..

[cit123] Paula S., Volkov A. G., Van Hoek A. N., Haines T. H., Deamer D. W. (1996). Permeation of protons, potassium ions, and small polar molecules through phospholipid bilayers as a function of membrane thickness. Biophys. J..

[cit124] Langecker M., Arnaut V., List J., Simmel F. C. (2014). DNA Nanostructures Interacting with Lipid Bilayer Membranes. Acc. Chem. Res..

[cit125] Douglas S. M., Marblestone A. H., Teerapittayanon S., Vazquez A., Church G. M., Shih W. M. (2009). Rapid prototyping of 3D DNA-origami shapes with caDNAno. Nucleic Acids Res..

[cit126] Langecker M., Arnaut V., Martin T. G., List J., Renner S., Mayer M. (2012). *et al.*, Synthetic Lipid Membrane Channels Formed by Designed DNA Nanostructures. Science.

[cit127] Xing Y., Dorey A., Jayasinghe L., Howorka S. (2022). Highly shape- and size-tunable membrane nanopores made with DNA. Nat. Nanotechnol..

[cit128] Thomsen R. P., Malle M. G., Okholm A. H., Krishnan S., Bohr S. S. R., Sørensen R. S. (2019). *et al.*, A large size-selective DNA nanopore with sensing applications. Nat. Commun..

[cit129] Diederichs T., Pugh G., Dorey A., Xing Y., Burns J. R., Hung Nguyen Q. (2019). *et al.*, Synthetic protein-conductive membrane nanopores built with DNA. Nat. Commun..

[cit130] Honig B., Nicholls A. (1995). Classical electrostatics in biology and chemistry. Science.

[cit131] Kowalczyk S. W., Wells D. B., Aksimentiev A., Dekker C. (2012). Slowing down DNA Translocation through a Nanopore in Lithium Chloride. Nano Lett..

[cit132] Keyser U. F., Koeleman B. N., van Dorp S., Krapf D., Smeets R. M. M., Lemay S. G. (2006). *et al.*, Direct force measurements on DNA in a solid-state nanopore. Nat. Phys..

[cit133] Boukhet M., Piguet F., Ouldali H., Pastoriza-Gallego M., Pelta J., Oukhaled A. (2016). Probing driving forces in aerolysin and α-hemolysin biological nanopores: electrophoresis versus electroosmosis. Nanoscale.

[cit134] Firnkes M., Pedone D., Knezevic J., Döblinger M., Rant U. (2010). Electrically Facilitated Translocations of Proteins through Silicon Nitride Nanopores: Conjoint and Competitive Action of Diffusion, Electrophoresis, and Electroosmosis. Nano Lett..

[cit135] Gu L. Q., Cheley S., Bayley H. (2003). Electroosmotic enhancement of the binding of a neutral molecule to a transmembrane pore. Proc. Natl. Acad. Sci. U. S. A..

[cit136] Huang G., Willems K., Soskine M., Wloka C., Maglia G. (2017). Electro-osmotic capture and ionic discrimination of peptide and protein biomarkers with FraC nanopores. Nat. Commun..

[cit137] Laohakunakorn N., Keyser U. F. (2015). Electroosmotic flow rectification in conical nanopores. Nanotechnology.

[cit138] Guo W., Tian Y., Jiang L. (2013). Asymmetric Ion Transport through Ion-Channel-Mimetic Solid-State Nanopores. Acc. Chem. Res..

[cit139] Piguet F., Discala F., Breton M. F., Pelta J., Bacri L., Oukhaled A. (2014). Electroosmosis through α-Hemolysin That Depends on Alkali Cation Type. J. Phys. Chem. Lett..

[cit140] Wong C. T. A., Muthukumar M. (2007). Polymer capture by electro-osmotic flow of oppositely charged nanopores. J. Chem. Phys..

[cit141] Verschueren D. V., Jonsson M. P., Dekker C. (2015). Temperature dependence of DNA translocations through solid-state nanopores. Nanotechnology.

[cit142] Meller A., Nivon L., Brandin E., Golovchenko J., Branton D. (2000). Rapid nanopore discrimination between single polynucleotide molecules. Proc. Natl. Acad. Sci. U. S. A..

[cit143] Franceschini L., Brouns T., Willems K., Carlon E., Maglia G. (2016). DNA Translocation through Nanopores at Physiological Ionic Strengths Requires Precise Nanoscale Engineering. ACS Nano.

[cit144] Willems K., Ruić D., Biesemans A., Galenkamp N. S., Van Dorpe P., Maglia G. (2019). Engineering and Modeling the Electrophoretic Trapping of a Single Protein Inside a Nanopore. ACS Nano.

[cit145] Sarabadani J., Ikonen T., Mökkönen H., Ala-Nissila T., Carson S., Wanunu M. (2017). Driven translocation of a semi-flexible polymer through a nanopore. Sci. Rep..

[cit146] Hu G., Xi G., Yan H., Gao Z., Wu Z., Lu Z. (2022). *et al.*, A molecular dynamics investigation of Taq DNA polymerase and its complex with a DNA substrate using a solid-state nanopore biosensor. Phys. Chem. Chem. Phys..

[cit147] Wen C., Schmid S., Dekker C. (2024). Understanding Electrophoresis and Electroosmosis in Nanopore Sensing with the Help of the Nanopore Electro-Osmotic Trap. ACS Nano.

[cit148] Wen C., Bertosin E., Shi X., Dekker C., Schmid S. (2023). Orientation-Locked DNA Origami for Stable Trapping of Small Proteins in the Nanopore Electro-Osmotic Trap. Nano Lett..

[cit149] Van Meervelt V., Soskine M., Singh S., Schuurman-Wolters G. K., Wijma H. J., Poolman B. (2017). *et al.*, Real-Time Conformational Changes and Controlled Orientation of Native Proteins Inside a Protein Nanoreactor. J. Am. Chem. Soc..

[cit150] Wolfenden R., Snider M. J. (2001). The Depth of Chemical Time and the Power of Enzymes as Catalysts. Acc. Chem. Res..

[cit151] Callender R., Dyer R. B. (2006). Advances in Time-Resolved Approaches To Characterize the Dynamical Nature of Enzymatic Catalysis. Chem. Rev..

[cit152] Delhommel F., Gabel F., Sattler M. (2020). Current approaches for integrating solution NMR spectroscopy and small-angle scattering to study the structure and dynamics of biomolecular complexes. J. Mol. Biol..

[cit153] East K. W., Skeens E., Cui J. Y., Belato H. B., Mitchell B., Hsu R. (2020). *et al.*, NMR and computational methods for molecular resolution of allosteric pathways in enzyme complexes. Biophys. Rev..

[cit154] Tsai M. D., Wu W. J., Ho M. C. (2022). Enzymology and Dynamics by Cryogenic Electron Microscopy. Annu. Rev. Biophys..

[cit155] Zernia S., van der Heide N. J., Galenkamp N. S., Gouridis G., Maglia G. (2020). Current Blockades of Proteins inside Nanopores for Real-Time Metabolome Analysis. ACS Nano.

[cit156] de Boer M., Gouridis G., Vietrov R., Begg S. L., Schuurman-Wolters G. K., Husada F. (2019). *et al.*, Conformational and dynamic plasticity in substrate-binding proteins underlies selective transport in ABC importers. eLife.

[cit157] van den Noort M., de Boer M., Poolman B. (2021). Stability of Ligand-induced Protein Conformation Influences Affinity in Maltose-binding Protein. J. Mol. Biol..

[cit158] Li X., Lee K. H., Shorkey S., Chen J., Chen M. (2020). Different Anomeric Sugar Bound States of Maltose Binding Protein Resolved by a Cytolysin A Nanopore Tweezer. ACS Nano.

[cit159] Hu R., Rodrigues J. V., Waduge P., Yamazaki H., Cressiot B., Chishti Y. (2018). *et al.*, Differential Enzyme Flexibility Probed Using Solid-State Nanopores. ACS Nano.

[cit160] Fierke C. A., Johnson K. A., Benkovic S. J. (1987). Construction and evaluation of the kinetic scheme associated with dihydrofolate reductase from Escherichia coli. Biochemistry.

[cit161] Panjarian S., Iacob R. E., Chen S., Engen J. R., Smithgall T. E. (2013). Structure and Dynamic Regulation of Abl Kinases*. J. Biol. Chem..

[cit162] Taylor S. S., Kornev A. P. (2011). Protein kinases: evolution of dynamic regulatory proteins. Trends Biochem. Sci..

[cit163] Kerns S. J., Agafonov R. V., Cho Y. J., Pontiggia F., Otten R., Pachov D. V. (2015). *et al.*, The energy landscape of adenylate kinase during catalysis. Nat. Struct. Mol. Biol..

[cit164] Aviram H. Y., Pirchi M., Mazal H., Barak Y., Riven I., Haran G. (2018). Direct observation of ultrafast large-scale dynamics of an enzyme under turnover conditions. Proc. Natl. Acad. Sci. U. S. A..

[cit165] Nam K., Thodika A. R. A., Tischlik S., Phoeurk C., Nagy T. M., Schierholz L. (2024). *et al.*, Magnesium induced structural reorganization in the active site of adenylate kinase. Sci. Adv..

[cit166] Pelz B., Žoldák G., Zeller F., Zacharias M., Rief M. (2016). Subnanometre enzyme mechanics probed by single-molecule force spectroscopy. Nat. Commun..

[cit167] Wolf-Watz M., Thai V., Henzler-Wildman K., Hadjipavlou G., Eisenmesser E. Z., Kern D. (2004). Linkage between dynamics and catalysis in a thermophilic-mesophilic enzyme pair. Nat. Struct. Mol. Biol..

[cit168] Henzler-Wildman K. A., Thai V., Lei M., Ott M., Wolf-Watz M., Fenn T. (2007). *et al.*, Intrinsic motions along an enzymatic reaction trajectory. Nature.

[cit169] Barber E. D., Bright H. J. (1968). The rate of an allosteric process: inhibition of homoserine dehydrogenase I from *E. coli* by threonine. Proc. Natl. Acad. Sci. U. S. A..

[cit170] Appleman J. R., Howell E. E., Kraut J., Blakley R. L. (1990). Role of aspartate 27 of dihydrofolate reductase from Escherichia coli in interconversion of active and inactive enzyme conformers and binding of NADPH. J. Biol. Chem..

[cit171] Penner M. H., Frieden C. (1985). Substrate-induced hysteresis in the activity of Escherichia coli dihydrofolate reductase. J. Biol. Chem..

[cit172] Behzadi A., Hatleskog R., Ruoff P. (1999). Hysteretic enzyme adaptation to environmental pH: change in storage pH of alkaline phosphatase leads to a pH-optimum in the opposite direction to the applied change. Biophys. Chem..

[cit173] Sha W., Moore J., Chen K., Lassaletta A. D., Yi C. S., Tyson J. J. (2003). *et al.*, Hysteresis drives cell-cycle transitions in Xenopus laevis egg extracts. Proc. Natl. Acad. Sci. U. S. A..

[cit174] Ferrell J. E. (2002). Self-perpetuating states in signal transduction: positive feedback, double-negative feedback and bistability. Curr. Opin. Cell Biol..

[cit175] Vinces T. C., de Souza A. S., Carvalho C. F., Visnardi A. B., Teixeira R. D., Llontop E. E. (2024). *et al.*, Monomeric Esterase: Insights into Cooperative Behavior, Hysteresis/Allokairy. Biochemistry.

[cit176] Jiang Y., Li X., Morrow B. R., Pothukuchy A., Gollihar J., Novak R. (2019). *et al.*, Single-Molecule Mechanistic Study of Enzyme Hysteresis. ACS Cent. Sci..

[cit177] Gilboa T., Ogata A. F., Reilly C. B., Walt D. R. (2022). Single-molecule studies reveal method for tuning the heterogeneous activity of alkaline phosphatase. Biophys. J..

[cit178] Gundersen L. E., Dunlap R. B., Harding N. G. L., Freisheim J. H., Otting F., Huennekens F. M. (1972). Dihydrofolate reductase from amethopterin-resistant Lactobacillus casei. Biochemistry.

[cit179] Huennekens F. M., Dunlap R. B., Freisheim J. H., Gundersen L. E., Harding N. G. L., Levison S. A. (1971). *et al.*, Dihydrofolate Reductases: Structural and Mechanistic Aspects*. Ann. N. Y. Acad. Sci..

[cit180] Perkins J. P., Hillcoat B. L. L., Bertino J. R. R. (1967). Dihydrofolate Reductase from a Resistant Subline of the L1210 Lymphoma. J. Biol. Chem..

[cit181] Dunn S. M. J., Batchelor J. G., King R. W. (1978). Kinetics of ligand binding to dihydrofolate reductase: binary complex formation with NADPH and coenzyme analogues. Biochemistry.

[cit182] Dunn S. M. J., King R. W. (1980). Kinetics of ternary complex formation between dihydrofolate reductase, coenzyme, and inhibitors. Biochemistry.

[cit183] Cayley P. J., Dunn S. M. J., King R. W. (1981). Kinetics of substrate, coenzyme, and inhibitor binding to Escherichia coli dihydrofolate reductase. Biochemistry.

[cit184] Chen J. T., Taira K., Tu C. P. D., Benkovic S. J. (1987). Probing the functional role of phenylalanine-31 of Escherichia coli dihydrofolate reductase by site-directed mutagenesis. Biochemistry.

[cit185] Zhang Z., Rajagopalan P. T. R., Selzer T., Benkovic S. J., Hammes G. G. (2004). Single-molecule and transient kinetics investigation of the interaction of dihydrofolate reductase with NADPH and dihydrofolate. Proc. Natl. Acad. Sci. U. S. A..

[cit186] Wei K. Y., Moschidi D., Bick M. J., Nerli S., McShan A. C., Carter L. P. (2020). *et al.*, Computational design of closely related proteins that adopt two well-defined but structurally divergent folds. Proc. Natl. Acad. Sci. U. S. A..

[cit187] Sakuma M., Honda S., Ueno H., Tabata K. V., Miyazaki K., Tokuriki N. (2023). *et al.*, Genetic Perturbation Alters Functional Substates in Alkaline Phosphatase. J. Am. Chem. Soc..

[cit188] St-Jacques A. D., Rodriguez J. M., Eason M. G., Foster S. M., Khan S. T., Damry A. M. (2023). *et al.*, Computational remodeling of an enzyme conformational landscape for altered substrate selectivity. Nat. Commun..

[cit189] Rago F., Saltzberg D., Allen K. N., Tolan D. R. (2015). Enzyme Substrate Specificity Conferred by Distinct Conformational Pathways. J. Am. Chem. Soc..

[cit190] Chen R., Gao B., Liu X., Ruan F., Zhang Y., Lou J. (2017). *et al.*, Molecular insights into the enzyme promiscuity of an aromatic prenyltransferase. Nat. Chem. Biol..

[cit191] Monod J., Changeux J. P., Jacob F. (1963). Allosteric proteins and cellular control systems. J. Mol. Biol..

[cit192] Motlagh H. N., Wrabl J. O., Li J., Hilser V. J. (2014). The ensemble nature of allostery. Nature.

[cit193] Tsai C. J., del Sol A., Nussinov R. (2009). Protein allostery, signal transmission and dynamics: a classification scheme of allosteric mechanisms. Mol. BioSyst..

[cit194] Cui Q., Karplus M. (2008). Allostery and cooperativity revisited. Protein Sci..

[cit195] Tzeng S. R., Kalodimos C. G. (2011). Protein dynamics and allostery: an NMR view. Curr. Opin. Struct. Biol..

[cit196] Gunasekaran K., Ma B., Nussinov R. (2004). Is allostery an intrinsic property of all dynamic proteins?. Proteins:Struct., Funct., Bioinf..

[cit197] Pan H., Lee J. C., Hilser V. J. (2000). Binding sites in Escherichia coli dihydrofolate reductase communicate by modulating the conformational ensemble. Proc. Natl. Acad. Sci. U. S. A..

[cit198] Nussinov R., Tsai C. J., Ma B. (2013). The Underappreciated Role of Allostery in the Cellular Network. Annu. Rev. Biophys..

[cit199] Tzeng S. R., Kalodimos C. G. (2012). Protein activity regulation by conformational entropy. Nature.

[cit200] Tzeng S. R., Kalodimos C. G. (2009). Dynamic activation of an allosteric regulatory protein. Nature.

[cit201] Schrank T. P., Bolen D. W., Hilser V. J. (2009). Rational modulation of conformational fluctuations in adenylate kinase reveals a local unfolding mechanism for allostery and functional adaptation in proteins. Proc. Natl. Acad. Sci. U. S. A..

[cit202] Hilser V. J., Thompson E. B. (2007). Intrinsic disorder as a mechanism to optimize allosteric coupling in proteins. Proc. Natl. Acad. Sci. U. S. A..

[cit203] Wright P. E., Dyson H. J. (1999). Intrinsically unstructured proteins: re-assessing the protein structure-function paradigm. J. Mol. Biol..

[cit204] Liu J., Perumal N. B., Oldfield C. J., Su E. W., Uversky V. N., Dunker A. K. (2006). Intrinsic Disorder in Transcription Factors. Biochemistry.

[cit205] Uversky V. N. (2011). Intrinsically disordered proteins from A to Z. Int. J. Biochem. Cell Biol..

[cit206] Liu Y., Pan T., Wang K., Wang Y., Yan S., Wang L. (2021). *et al.*, Allosteric Switching of Calmodulin in a Mycobacterium smegmatis porin A (MspA) Nanopore-Trap. Angew. Chem., Int. Ed..

[cit207] Ma H., Wang Y., Li Y. X., Xie B. K., Hu Z. L., Yu R. J. (2024). *et al.*, Label-Free Mapping of Multivalent Binding Pathways with Ligand–Receptor-Anchored Nanopores. J. Am. Chem. Soc..

[cit208] Derrington I. M., Butler T. Z., Collins M. D., Manrao E., Pavlenok M., Niederweis M. (2010). *et al.*, Nanopore DNA sequencing with MspA. Proc. Natl. Acad. Sci. U. S. A..

[cit209] Derrington I. M., Craig J. M., Stava E., Laszlo A. H., Ross B. C., Brinkerhoff H. (2015). *et al.*, Subangstrom single-molecule measurements of motor proteins using a nanopore. Nat. Biotechnol..

[cit210] Wendell D., Jing P., Geng J., Subramaniam V., Lee T. J., Montemagno C. (2009). *et al.*, Translocation of double-stranded DNA through membrane-adapted phi29 motor protein nanopores. Nat. Nanotechnol..

[cit211] Wang S., Haque F., Rychahou P. G., Evers B. M., Guo P. (2013). Engineered Nanopore of Phi29 DNA-Packaging Motor for Real-Time Detection of Single Colon Cancer Specific Antibody in Serum. ACS Nano.

[cit212] Huang G. , Versloot R. C. A. and Maglia G., Detection of single amino acid differences in haemoglobin from blood samples using a nanopore, 2021 10.1002/anie.202206227 PMC9541544 35759385

[cit213] Huang G., Willems K., Bartelds M., van Dorpe P., Soskine M., Maglia G. (2020). Electro-Osmotic Vortices Promote the Capture of Folded Proteins by PlyAB Nanopores. Nano Lett..

[cit214] Bräuning B., Bertosin E., Praetorius F., Ihling C., Schatt A., Adler A. (2018). *et al.*, Structure and mechanism of the two-component α-helical pore-forming toxin YaxAB. Nat. Commun..

[cit215] Schubert E., Vetter I. R., Prumbaum D., Penczek P. A., Raunser S. (2018). Membrane insertion of α-xenorhabdolysin in near-atomic detail. eLife.

[cit216] Zhang S., Cao Z., Fan P., Wang Y., Jia W., Wang L. (2022). *et al.*, A Nanopore-Based Saccharide Sensor. Angew. Chem., Int. Ed..

[cit217] Spitzer J. J., Poolman B. (2005). Electrochemical structure of the crowded cytoplasm. Trends Biochem. Sci..

[cit218] Xiang L., Yan R., Chen K., Li W., Xu K. (2023). Single-Molecule Displacement Mapping Unveils Sign-Asymmetric Protein Charge Effects on Intraorganellar Diffusion. Nano Lett..

[cit219] Rincon-Restrepo M., Mikhailova E., Bayley H., Maglia G. (2011). Controlled Translocation of Individual DNA Molecules through Protein Nanopores with Engineered Molecular Brakes. Nano Lett..

[cit220] Stępień P., Świątek S., Robles M. Y. Y., Markiewicz-Mizera J., Balakrishnan D., Inaba-Inoue S. (2023). *et al.*, CRAFTing Delivery of Membrane Proteins into Protocells using Nanodiscs. ACS Appl. Mater. Interfaces.

[cit221] Rauh O. , Kukovetz K., Winterstein L., Introini B. and Thiel G., *Combining in vitro translation with nanodisc technology and functional reconstitution of channels in planar lipid bilayers*, 2021, pp. 293–318 10.1016/bs.mie.2021.02.003 34059286

[cit222] Conti Nibali S., Di Rosa M. C., Rauh O., Thiel G., Reina S., De Pinto V. (2021). Cell-free electrophysiology of human VDACs incorporated into nanodiscs: An improved method. Biophys. Rep..

[cit223] Tanaka K., Caaveiro J. M. M., Morante K., González-Manãs J. M., Tsumoto K. (2015). Structural basis for self-assembly of a cytolytic pore lined by protein and lipid. Nat. Commun..

[cit224] De Colibus L., Sonnen A. F. P., Morris K. J., Siebert C. A., Abrusci P., Plitzko J. (2012). *et al.*, Structures of Lysenin Reveal a Shared Evolutionary Origin for Pore-Forming Proteins And Its Mode of Sphingomyelin Recognition. Structure.

[cit225] Sakurai N., Kaneko J., Kamio Y., Tomita T. (2004). Cloning, expression, and pore-forming properties of mature and precursor forms of pleurotolysin, a sphingomyelin-specific two-component cytolysin from the edible mushroom Pleurotus ostreatus. Biochim. Biophys. Acta, Gene Struct. Expression.

[cit226] Vreeker E. , Grünewald F., van der Heide N. J., Marrink S. J., Tych (Kasia) K. and Maglia G., Hybrid lipid-block copolymer membranes enable stable reconstitution of a wide range of nanopores and robust sampling of serum, 2024

[cit227] Hall A. R., Scott A., Rotem D., Mehta K. K., Bayley H., Dekker C. (2010). Hybrid pore formation by directed insertion of α-haemolysin into solid-state nanopores. Nat. Nanotechnol..

[cit228] Sen P., Hoi H., Gupta M. (2021). Low Noise Hybrid Nanopore with Engineered OmpG and Bilayer MoS 2. ACS Appl. Bio Mater..

[cit229] Cressiot B., Greive S. J., Mojtabavi M., Antson A. A., Wanunu M. (2018). Thermostable virus portal proteins as reprogrammable adapters for solid-state nanopore sensors. Nat. Commun..

[cit230] Cressiot B., Greive S. J., Si W., Pascoa T. C., Mojtabavi M., Chechik M. (2017). *et al.*, Porphyrin-Assisted Docking of a Thermophage Portal Protein into Lipid Bilayers: Nanopore Engineering and Characterization. ACS Nano.

[cit231] Mojtabavi M., Greive S. J., Antson A. A., Wanunu M. (2022). High-Voltage Biomolecular Sensing Using a Bacteriophage Portal Protein Covalently Immobilized within a Solid-State Nanopore. J. Am. Chem. Soc..

[cit232] Yusko E. C., Bruhn B. R., Eggenberger O. M., Houghtaling J., Rollings R. C., Walsh N. C. (2017). *et al.*, Real-time shape approximation and fingerprinting of single proteins using a nanopore. Nat. Nanotechnol..

[cit233] Ma H., Yu R. J., Ying Y. L., Long Y. T. (2022). Electrochemically confined effects on single enzyme detection with nanopipettes. J. Electroanal. Chem..

[cit234] Li W., Zhou J., Lan Q., Ding X. L., Pan X. T., Ahmed S. A. (2023). *et al.*, Single-Molecule Electrical and Spectroscopic Profiling Protein Allostery Using a Gold Plasmonic Nanopore. Nano Lett..

[cit235] Wei R., Gatterdam V., Wieneke R., Tampé R., Rant U. (2012). Stochastic sensing of proteins with receptor-modified solid-state nanopores. Nat. Nanotechnol..

[cit236] Li Q., Ying Y. L., Liu S. C., Lin Y., Long Y. T. (2019). Detection of Single Proteins with a General Nanopore Sensor. ACS Sens..

[cit237] Liu S. C., Ying Y. L., Li W. H., Wan Y. J., Long Y. T. (2021). Snapshotting the transient conformations and tracing the multiple pathways of single peptide folding using a solid-state nanopore. Chem. Sci..

[cit238] Schmid S., Dekker C. (2021). The NEOtrap – en route with a new single-molecule technique. iScience.

[cit239] Wan Y. K., Hendra C., Pratanwanich P. N., Göke J. (2022). Beyond sequencing: machine learning algorithms extract biology hidden in Nanopore signal data. Trends Genet..

[cit240] Greive S. J., Bacri L., Cressiot B., Pelta J. (2024). Identification of Conformational Variants for Bradykinin Biomarker Peptides from a Biofluid Using a Nanopore and Machine Learning. ACS Nano.

[cit241] Dutt S., Shao H., Karawdeniya B., Bandara Y. M. N. D. Y., Daskalaki E., Suominen H. (2023). *et al.*, High Accuracy Protein Identification: Fusion of Solid-State Nanopore Sensing and Machine Learning. Small Methods.

[cit242] Lucas F. L. R., Piso T. R. C., van der Heide N. J., Galenkamp N. S., Hermans J., Wloka C. (2021). *et al.*, Automated Electrical Quantification of Vitamin B1 in a Bodily Fluid using an Engineered Nanopore Sensor. Angew. Chem., Int. Ed..

[cit243] Comstock M. J., Ha T., Chemla Y. R. (2011). Ultrahigh-resolution optical trap with single-fluorophore sensitivity. Nat. Methods.

[cit244] Cai S., Sze J. Y. Y., Ivanov A. P., Edel J. B. (2019). Small molecule electro-optical binding assay using nanopores. Nat. Commun..

[cit245] Ivankin A., Henley R. Y., Larkin J., Carson S., Toscano M. L., Wanunu M. (2014). Label-Free Optical Detection of Biomolecular Translocation through Nanopore Arrays. ACS Nano.

[cit246] Li S., Zeng S., Wen C., Zhang Z., Hjort K., Zhang S. L. (2022). Docking and Activity of DNA Polymerase on Solid-State Nanopores. ACS Sens..

[cit247] Hemmig E. A., Fitzgerald C., Maffeo C., Hecker L., Ochmann S. E., Aksimentiev A. (2018). *et al.*, Optical Voltage Sensing Using DNA Origami. Nano Lett..

[cit248] Fried J. P., Wu Y., Tilley R. D., Gooding J. J. (2022). Optical Nanopore Sensors for Quantitative Analysis. Nano Lett..

